# ﻿Long-distance gene flow and recombination shape the evolutionary history of a maize pathogen

**DOI:** 10.3897/imafungus.16.138888

**Published:** 2025-02-21

**Authors:** Flávia Rogério, Cock Van Oosterhout, Stéphane De Mita, Francisco Borja Cuevas-Fernández, Pablo García-Rodríguez, Sioly Becerra, Silvia Gutiérrez-Sánchez, Andrés G. Jacquat, Wagner Bettiol, Guilherme Kenichi Hosaka, Sofia B. Ulla, Jürg Hiltbrunner, Rogelio Santiago, Pedro Revilla, José S. Dambolena, José L. Vicente-Villardón, Ivica Buhiniček, Serenella A. Sukno, Michael R. Thon

**Affiliations:** 1 Department of Microbiology and Genetics, Institute for Agribiotechnology Research (CIALE), University of Salamanca, Villamayor, Salamanca, Spain; 2 Present Address: Department of Plant Pathology, University of Florida, Gainesville, Florida 32611, USA; 3 School of Environmental Sciences, University of East Anglia, Norwich Research Park, Norwich, UK; 4 PHIM Plant Health Institute, Univ Montpellier, INRAE, CIRAD, Institut Agro, IRD, Montpellier, France; 5 Faculty of Exact, Physical and Natural Science, National University of Córdoba, IMBIV-CONICET-ICTA, Córdoba, Argentina; 6 Embrapa Environment, Jaguariúna, São Paulo, Brazil; 7 Laboratory of Genetics of Microorganisms “Prof. Joao Lucio de Azevedo”, Department of Genetics, “Luiz de Queiroz” College of Agriculture, University of São Paulo, Piracicaba, Brazil; 8 Federal Department of Economic Affairs, Agroscope, Centre of Competences Plants and Plant Products, Zurich, Switzerland; 9 Misión Biológica de Galicia, Spanish National Research Council (CSIC), Pontevedra, Spain; 10 Statistics Department, University of Salamanca, Salamanca, Spain; 11 Bc Institute for Breeding and Production of Field Crops, Dugo Selo, Croatia

**Keywords:** *Colletotrichumgraminicola*, genetic introgression, isolation by distance (IBD), population genomics, restriction site-associated DNA sequencing (RAD-seq), whole-genome sequencing (WGS)

## Abstract

The evolutionary history of crop pathogens is shaped by a complex interaction of natural and anthropogenic factors. The fungus *Colletotrichumgraminicola* causes maize anthracnose which results in significant yield losses worldwide. We conducted a comprehensive investigation into the evolutionary genomics of *C.graminicola* using a collection of 212 isolates from 17 countries across five continents. Genomic analyses supported the existence of three geographically isolated genetic lineages, with a significant pattern of isolation by distance. We identified two distinct gene flow patterns, driven by short- and long-distance dispersal, likely resulting from the natural spread of the pathogen and the exchange of contaminated seeds. We present evidence of genetic introgression between lineages, suggesting a long history of recombination. We identified significant recombination events coalescing at distinct points in time, with the North American lineage displaying evidence of the most ancient recombination. Demographic modelling has indicated that North America is an intermediate between Brazil, Europe and an ancestral, unsampled source population, which is hypothesised to be Mesoamerican. Our analyses revealed that the global genomic structure of *C.graminicola* is shaped by geographic differentiation driven by long-distance migration and a long history of recombination and introgression. We show historical relationships amongst these lineages, identifying a potential route for fungal spread, with the North American population emerging ancestrally, followed sequentially by the Brazilian and European populations. Our research indicates that the European lineage is more virulent, which has implications for the potential emergence of new outbreaks of maize anthracnose in Europe.

## ﻿Introduction

Examining the processes that drive species evolution over time is a central focus of evolutionary genomics. Population genetics provides valuable insights into patterns of genetic variation within populations, enhancing our comprehension of their evolutionary history and dynamics (Dobzhansky 1973; Gladieux et al. 2011; Casillas and Barbadilla 2017). This knowledge has practical applications for studying how populations of plant pathogens respond to future environmental changes and selective pressure or for determining the role that human activities play in the dispersion of pathogens, which is crucial for effective disease control (Giraud et al. 2008; McDonald and Linde 2002; Everhart et al. 2021).

The genetic composition of pathogen populations is shaped by evolutionary forces and interactions with hosts and local the environmental conditions (Charlesworth 2010). Anthropogenic changes to the environment have a large impact on the interactions between evolutionary forces, which, in turn, have altered the Red Queen Dynamics between parasites and their hosts (Van Oosterhout 2021). Migration encompasses individual or propagule movement, leading to gene flow between isolated gene pools (McDermott and McDonald 1993). Understanding migration patterns is vital for providing information for disease prevention decisions, as it may introduce new genetic variations, including virulence factors and connect adapted populations (McDonald and Linde 2002). Together with gene flow, genetic recombination constitutes a vital evolutionary force driving variation and pathogen adaptation (Stukenbrock and Bataillon 2012; Milgroom 2015; de Vries et al. 2020). Genetic recombination has the potential to accelerate adaptation by the generation of new, potentially adaptive genotypes that are favoured by natural selection or by the combination of adaptive alleles at distinct loci that appear in different genetic backgrounds (Barton 2010). This process offers new substrates for adaptive evolution while simultaneously purging the genome of deleterious mutations accumulated over time (Felsenstein 1974; Taylor et al. 1999; Van Oosterhout 2021).

The fungus *Colletotrichumgraminicola* is an important model for studying plant-pathogen interactions, being the pioneer amongst *Colletotrichum* species for genome sequencing (Perfect et al. 1999; O’Connell et al. 2012; Becerra et al. 2023). The genetic structure of *C.graminicola* has been explored globally (Rogério et al. 2023). Population genomics analyses have revealed that the pathogen is structured into three largely isolated populations associated with North America, Europe and Brazil. Genetic exchanges between these populations are facilitated by intra- and intercontinental migration, with evidence of genetic recombination. Nevertheless, the current impact of gene flow and recombination at the genome level, as well as the evolutionary origin of these populations, remains unknown.

Population genomics studies of many crop pathogens have demonstrated that human activities (domestication, trade and migration) play pivotal roles in the emergence and spread of plant diseases (Barrès et al. 2008; Chakraborty 2013; Goss et al. 2014; Kamvar et al. 2015b; McMullan et al. 2018; Tabima et al. 2020; Sotiropoulos et al. 2022). The dissemination of new virulence genes and agrochemical resistance amongst populations across large geographical areas is significantly influenced by gene flow, which plays a central role in driving pathogen evolution (Zhan et al. 2015). Like mutation and recombination, gene flow is a source of new genetic variation, which forms the substrate for selection in host-parasite interactions. By introducing novel alleles from other populations, gene flow indirectly increases the effective population sizes of the pathogen. The increased rates of gene flow in our human-modified environment give present-day pathogens an important co-evolutionary advantage (Van Oosterhout 2021). Understanding and monitoring gene flow is crucial for developing effective strategies to manage and mitigate the potential associated risks. Competition theory posits that a reduction in the competition of related genotypes may lead to the selection of more aggressive strains (Koskella et al. 2006; López-Villavicencio et al. 2007; Zhan and McDonald 2013). In this context, gene flow between genetically divergent lineages can decrease genetic relatedness and potentially promote the emergence of these more aggressive strains. This can pose a risk factor for host resistance breakdown.

Fungi exhibit a range of reproductive strategies involving genetic recombination outside conventional sexual reproduction, such as hyphal fusion (anastomosis) and parasexual cycles (Giraud et al. 2008). While clonality is the predominant mode of reproduction, population genetics studies highlight that recombination plays a key role in the genetic structure of some phytopathogenic fungi (Roca et al. 2005; Meng et al. 2015; Taylor et al. 2015; Ashu et al. 2017; Stukenbrock and Dutheil 2018; Drenth et al. 2019). Notably, recombination is reported to be an important factor in the ecology and evolutionary dynamics of numerous *Colletotrichum* species (Gale 2003; Souza-Paccola et al. 2003; Crouch et al. 2006; Diao et al. 2015; Rogério et al. 2023). However, certain studies have elucidated alternative mechanisms, such as the parasexual cycle, contributing to genetic recombination in the *Colletotrichum* genus (Souza-Paccola et al. 2003; Rosada et al. 2010; Barcelos et al. 2014; Nordzieke et al. 2019).

Gene flow and recombination lead to genomes with admixed ancestry, a process that is commonly referred to as genetic admixture (Beichman et al. 2018). The genomic signature of admixture is characterised by chromosomal segments that originate from distinct genetic backgrounds or populations (Carroll 2013). Closely affiliated with admixture is the term “introgression”. Introgression is a process resulting from recombination and gene flow between significantly divergent gene pools, leading to the gradual transfer of genetic material from one pool to the other through admixed individuals (Anderson 1953; Stukenbrock 2016). At the far end of this continuum is horizontal gene transfer, which is the genetic exchange between otherwise reproductively isolated species. In this study, we adopted the term introgression to refer to genetic exchanges between divergent lineages. These genetic exchanges leave distinct signatures in the genome, identified through DNA sequence comparisons, with introgressed regions typically exhibiting high nucleotide similarity, followed by a sudden change in divergence (Gompert and Buerkle 2013; Rosenzweig et al. 2016; Ward and van Oosterhout 2016).

In this study, we focused on unravelling the evolutionary dynamics shaping the genetic structure of *C.graminicola*. Specifically, we addressed key questions: What drives the genetic structure in *C.graminicola* populations? What role does genetic introgression play in its evolutionary history? What are the sources and historical relationships of its lineages? Our investigation employed a global collection of 212 isolates from 17 countries, enabling a comprehensive analysis of genetic exchanges at the genome scale and determination of the evolutionary origin of *C.graminicola* lineages. By addressing these questions, our study contributes to a deeper understanding of the evolutionary genomics of *C.graminicola*, with implications for disease management.

## ﻿Methods

### ﻿Data processing

In total, 212 isolates of *C.graminicola* were obtained from field samples and public culture collections from seventeen countries (Suppl. material 1: table S1). Mycelia from monosporic cultures were incubated in an orbital shaker in potato dextrose broth (PDB), for 3 days at 25 °C and 150 rpm under continuous light. Genomic DNA was extracted using DNeasy Plant Mini Kits (Qiagen Inc., Valencia, California, USA) according to manufacturer’s instructions. Ninety-four isolates from Rogério et al. (2023) were included, along with 118 new isolates, sequenced using restriction site-associated DNA sequencing (RAD-seq) or whole-genome sequencing (WGS). A total of 82 new isolates were genotyped with RAD-seq by Floragenex, Inc. (﻿Beaverton, OR, USA). RAD-seq libraries with sample-specific barcode sequences were constructed from DNA digested with the restriction enzyme PstI and then single-end sequenced (1 × 100 bp) in one lane of an Illumina HiSeq 2000 instrument (San Diego, CA; Beaverton, OR, USA). Whole-genome resequencing of 36 isolates was performed by Beijing Genomics Institute (Hong Kong, China) via the DNBSEQ library sequencing platform (2 × 150 bp).

The RAD-seq reads were demultiplexed and quality filtered via the process_radtags module of the software Stacks v.2.10 (Rochette et al. 2019), using default parameters. The demultiplexed reads were aligned to the *C.graminicola* reference genome M1.001 (NCBI accession number PRJNA900520) with BWA v.0.7.8 (Li and Durbin 2009). For the WGS reads, the sequence quality was checked with fastqc v.0.11.7 (Babraham Bioinformatics) and low-quality bases with Phred score of less than 30 were trimmed with trimmomatic v.0.3.8 (Bolger et al. 2014). The alignments were sorted and each bam file was assigned to a read group containing metadata, such as sample name, library and platform information, using samtools v. 1.3 (Li et al. 2009). Variant calling was performed with the HaplotypeCaller module of gatk v.3.7, applying hard filters as suggested in GATK’s Best Practices (Van der Auwera et al. 2013). Additional filters were applied via VCFtools (Danecek et al. 2011), to retain ﻿only biallelic SNPs with a genotype rate of 90% and sites with a minor allele frequency of less than 5% were retained.

### ﻿Global population structure

Repeated multilocus genotypes (here referred to as clones) were identified via the function mlg.filter with a threshold determined via the cutoff_predictor tool on the basis of Euclidean distance via the package poppr v.2.8.6 (﻿Kamvar et al. 2015). Population genetic analyses were conducted on a clone-corrected dataset (i.e. only one representative of each repeated multilocus genotype was retained). To investigate population subdivision, first, we used the clustering method implemented in the snmf software to infer individual ancestry coefficients. The best number of clusters (*K*) was chosen using the cross-entropy criterion, based on the prediction of masked haplotypes to evaluate the error of ancestry estimation and the quality of fit of the model to the data. We ran 10 repetitions for each value of *K* ranging from 1 to 10. To confirm the population differentiation, we employed a principal component analysis (PCA), with *a priori* geographical knowledge from sampling (by continent) and a discriminate analysis of principal components (DAPC), both of which were conducted in R with the package ADEGENET v.2.0.1 (Jombart and Ahmed 2011). The find.cluster function was used to identify the most likely grouping in the data on the basis of the Bayesian Information Criterion (BIC) calculated for 1–10 clusters. Additionally, we reconstructed a neighbour network using splitstree v4 (Steinrücken et al. 2019) to visualise phylogenetic signals based on a distance matrix with default parameters.

To further understand the impact of geographic subdivision on population structure, we conducted isolation-by-distance analyses. We computed pairwise genetic dissimilarity and geographical distance between samples and examined their relationships via regression analysis. The correlation between the matrices of genetic and geographic distance was also observed via a simple Mantel test with 1000 permutations, as implemented in the vegan package in R (v.2.6.4). Genome variability was assessed through nucleotide diversity statistics computed via egglib v.3.3.2 (Siol et al. 2022).

### ﻿Footprints of recombination

To examine the impact of genetic exchanges on tree topologies, we constructed a consensus tree using densitree 2 (Bouckaert 2010). beauti v.1.5.3 was used to generate XML files for each contig. The substitution models were selected using bmodeltest (Bouckaert and Drummond 2017), which detects the best model and automatically adds it to the XML file. BEAST 2 (v.2.5.1) (Bouckaert et al. 2019) was used to generate phylogenetic trees via Markov Chain Monte Carlo (MCMC) for 1 × 10^6^ generations with a sample frequency of 2,000 and burn-in of 10%.

Recombination was analysed in multiple genome alignments by chromosome, including non-variant sites. We used the module genotypegvcs in GATK to include genotypes from all positions present in the reference genome and bcftools v.10.2 (Danecek et al. 2021) to obtain consensus sequences for each chromosome. Recombination events were analysed by the rdp4 software (Martin et al. 2021) via seven independent algorithms (rdp, geneconv, bootscan, maxchi, chimera, siscan and 3 seq). Significant events (*P* < 0.05) were considered if they were significant with three or more detection methods (Jouet et al. 2015). Only events for which the software identified the parental sequences (i.e. no “unknowns”) were considered. ﻿To visualise the significant recombinant blocks identified by rdp4, we used hybridcheck software (Ward and van Oosterhout 2016) with a step size of 1 and a window size of 500 SNPs. Hybridcheck was also used to date the recombination events. The divergence times between the donor and recombinant sequences were calculated via JC69 mutation correction, assuming a mutation rate of 10^−8^ per generation and a generation time of one year (Kasuga et al. 2002).

Recombination events were also dated through Bayesian Inference in Beast 2. We selected some recombinant blocks detected by hybridcheck and applied a Bayesian coalescent approach for dating. XML files were created using beauti, where the module bmodeltest was used to determine the optimal substitution model under a mutation rate of 10^−8^ and a strict clock rate. We assumed a model of a coalescent constant population with default priors and a chain length of 1 × 10^7^ generations. Each dating analysis was replicated five times. trace was used for visualising posterior parameters. The tree height statistic, which represents the marginal posterior distribution of the age of the root of the entire tree, was considered the divergence time from the common ancestor (tMRCA) for the set of sequences evaluated.

For dating, we assumed a generation time of 1 year. Note that the reported dates are likely an overestimate of the true age of recombination. In other words, the “true” recombination event may be more recent than our inferred estimate. There are two ways dating estimates can be biased, leading to overestimating the true date of introgression. First, there is an overestimation because the descendants of the actual donor and recipient sequences are unlikely to have been sampled.

Consequently, the introgressed regions look more divergent (and thus “older”). Second, even if the direct descendants of the donor sequence were included in our analysis, they may have diverged from the block in the recipient block owing to subsequent recombination. This may have introduced multiple nucleotide polymorphisms into the introgressed region. Given that we assume that divergence is driven by the mutation rate (i.e. a molecular clock), polymorphisms introduced by recombination would make the divergence appear older (see Jouet et al. (2015)). Both processes lead to an overestimation of the true date of introgression.

To characterise genes present in the introgressed regions, we extracted the transcripts and proteins from the corresponding regions in the reference genome (Becerra et al. 2023) using gffread (Pertea and Pertea 2020). For the enrichment analysis the Gene Ontology terms were assigned from re-annotated transcripts using blast2go (Götz et al. 2008) against the SwissProt database and used as input to the topgo R package (Alexa and Rahnenfuhrer 2021). We used signalp v. 5.0 (Almagro Armenteros et al. 2019) to identify secreted proteins.

### ﻿Demographic inference

We inferred the evolutionary history of *C.graminicola* genetic lineages using Approximate Bayesian Computation (ABC), based on random forest (RF), conducted in the Python package Pyabcranger (Collin et al. 2020). We based our analysis on three genetic lineages (North American, Brazilian and European) treated as independent populations. We used the Python package egglib to perform coalescent simulations and to compute the summary statistics and Pyabcranger to perform model choice and parameter estimation. ABC-RF uses a classification vote system after bagging (i.e. aggregating bootstrap results) the simulated outputs to identify the most likely model. The model with the highest number of votes ﻿is deemed to be the most likely model. To shed light on divergence events and colonisation routes, we modelled 22 scenarios of divergence, including asymmetrical migration and bottleneck events, assuming either stepping-stone or single-source models. Models with and without an unknown population (i.e. the ‘ghost’ population) as an ancestral population were tested. In our models, every divergence event was immediately followed by a bottleneck; otherwise, a constant population size was maintained for all populations. For the detailed methodology, see Suppl. material 2.

### ﻿Pathogenicity assays

Pathogenicity assays were performed by *in vivo* leaf blight assays, following Vargas et al. 2012), as described by Rogério et al. (2023). A total of 56 isolates were evaluated with ﻿three biological replicates, with three plants inoculated with each fungal strain. The average lesion size for each strain was measured by scanning leaves and quantifying the lesion size with the image processing software Paint.NET v.3.5 (https://www.getpaint.net). Due to the limitations of accommodating all the isolates simultaneously, assays were conducted in batches of 7–11 strains. The reference strain M1.001 was included in all batches to estimate batch effects. We compared the results of strain M1.001 across different batches via analysis of variance (ANOVA), with lesion size as the response variable, batch size as the predictor variable and the null hypothesis of no difference between batches. The highly significant differences (*P* < 0.0001) indicated a clear batch effect. To mitigate these effects, lesion sizes were standardised using the mean and standard deviation of the control strain specific to its batch. ANOVA of standardised data revealed significant differences amongst isolates, so we performed a Sidäk comparison-of-means test. This choice was made because it is exact for independent comparisons and easily translated into a graphical representation, facilitating the identification of significant differences amongst strains.

Additionally, the isolates tested in the virulence assay were divided into groups, based on sampling year and genetic structure. The isolates were classified into three categories, based on genetic group and virulence (less, equal or more virulent than isolate M1.001) and subjected to Fisher’s exact test. The isolates were also grouped by year into three categories (before 2012, between 2012 and 2016 and after 2016) and subjected to a Kruskal–Wallis test. The statistical tests and figures were implemented in the R.

Finally, we examined whether there was a positive correlation between pairwise genetic distance between isolates and similarity in their level of virulence. Genetic distance was calculated as the difference in the number of bases in pairwise comparisons. The similarity in virulence level was assessed by comparing the difference between lesion areas (calculated as the lesion area of isolate A minus the lesion area of isolate B). The correlation between pairwise genetic distance and similarity in virulence was analysed using a Mantel test with 1000 permutations, as implemented in the vegan package in R (v.2.6.4).

### ﻿Abbreviations

**RAD-Seq** Restriction site-associated DNA sequencing

**WGS** Whole Genome Shotgun Sequencing

NA North American

**EU** European

**BR** Brazilian

**AMOVA** Analysis of Molecular Variance

## ﻿Results

### ﻿Distinct levels of genetic diversity and admixture define *C.graminicola* lineages

**Figure 1.**
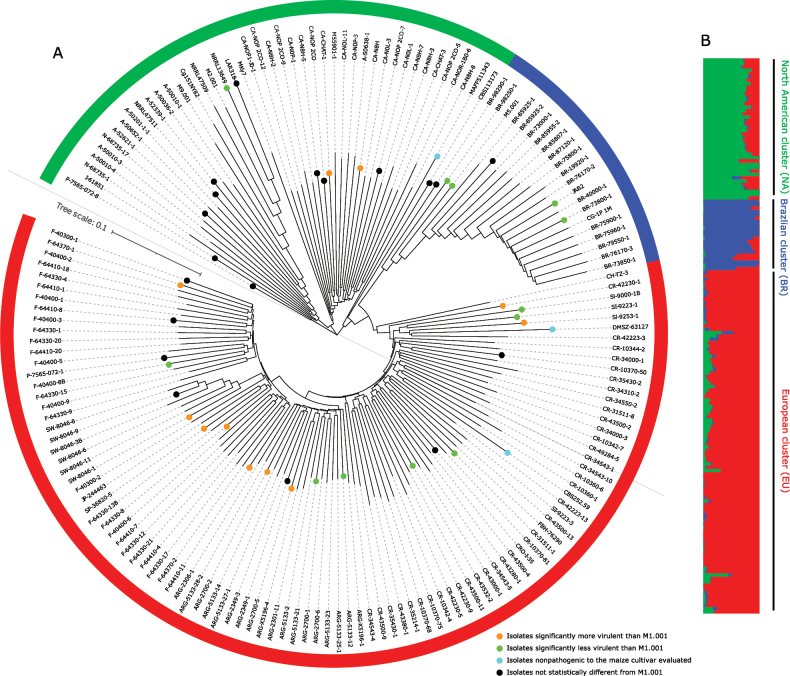
Population subdivision of *Colletotrichumgraminicola* based on 7,207 single-nucleotide polymorphisms in the clone-corrected dataset. **A** Neighbour-net network showing relationships between isolates, visualised in a circular tree via itol v. 6 (Letunic and Bork 2007). The dots represent isolates whose pathogenicity was essential. Samples SW-8046-2, P-7565-072-6, SW-8046-13, CR-49298-1, SL-9253-4, CRO-I-41, P-7565-072-3, CR-10342-5, F-64330-7 and F-64330-2 were removed from the network because they are clones; **B** individual ancestry proportions in *K* = 3 clusters estimated with snmf each genotype is represented by a vertical bar.

We characterised the global genetic structure of *C.graminicola* via a total of 212 isolates, based on 7,207 biallelic SNPs were distributed across 13 chromosomes, with an average of 1.3 SNPs per 10 kb (Suppl. material 3: fig. S1; Suppl. material 1: table S1). After clone correction, 166 unique multilocus genotypes were retained in the dataset (Suppl. material 1: table S2). A neighbour-net network clearly revealed three distinct genetic groups, with isolates largely grouped by their geographic origin: South America, Europe (including Argentina) and North America (Fig. 1A; Suppl. material 3: fig. S2). The observed population subdivision corroborates the conclusion drawn in our previous study (Rogério et al. 2023), which was based on a smaller number of isolates (Fig. 1. 108 versus 212 presently). By including more genetic variants, we obtained a better resolution of the genetic diversity distribution, but clustering methods still supported the existence of these groups. These three groups are referred to as Brazilian (BR), European (EU) and North American (NA) groups, respectively.

Population substructuring and admixture were analysed in further detail using the non-parametric method implemented in snmf. On the basis of the cross-entropy criterion, which was confirmed by the Bayesian Information Criterion, the model with *K* = 3 clusters best captures the population substructure of the whole dataset (Suppl. material 3: fig. S2). Interestingly, 80% of the isolates present evidence of admixture, which suggests genetic exchanges between *C.graminicola* genetic lineages (Fig. 1B; Suppl. material 1: table S4). Some isolates are highly admixed, such as P-7565-072-8, MAFF511343, CBS113173, M5.001, BR-98290-1 and BR-98250-1. These isolates are responsible for the “loops” or reticulate nodes in the neighbour network (Suppl. material 3: fig. S3). In contrast, a small proportion of isolates are completely pure, i.e. 20% of isolates have individual ancestry coefficients that assign them to a single cluster (*q* > 0.99, where *q* represents the proportion of an individual genome originating from a given ancestral gene pool) and are considered pure (Suppl. material 3: fig. S3, Suppl. material 1: table S4). The Brazilian cluster presented the largest proportion of pure isolates. The North American and Brazilian lineages showed the most ancestry shared with Europe, with isolates showing membership lower than 1% with other clusters (Suppl. material 1: table S4). There was one exception, i.e. the isolate MAFF511343, which originated from a public culture collection. Interestingly, older North American isolates are genetically pure: LAR318 (1990), M2.001 (1975), Cg151NY82 (1982), M9.001 (1990), NRLL13649 (1988) and NRLL47509 (2005). In addition, the NA lineage has the deepest rooting branch and these isolates have the longest terminal branches, indicating a more ancient origin of this group. The European clade encompasses all samples from European countries, one isolate from China and Japan and all isolates from Argentina (see highlighted samples in Suppl. material 3: figs S3, S4). Except for some Portuguese samples (P-7565-072-4, P-7565-072-6, P-7565-072-7, P-7565-072-8), all the isolates were grouped with samples from the same country of origin.

The relationships amongst all the genotypes visualised in a neighbour phylogenetic network (Suppl. material 3: fig. S3) supported a clear worldwide subdivision into three lineages, with long branches separating lineages by geographic origin. We observed several loops (or reticulations) in the network, indicating phylogenetic inconsistencies, where part of the focal sequence resembled that of a second sequence and one part of that sequence was more like a third sequence. This pattern is likely caused by recombination, as evidenced by the pairwise homoplasy index (PHI test), which was also calculated using splitstree which detected statistically significant evidence for recombination for the three lineages (*P* < 0.001).

The consensus tree generated by densitree, based on 1291 SNPs on chromosome 1, revealed many different topologies. This representation further corroborated the inferred population subdivision into three groups, with individuals from different lineages consistently separated by consensus branches (Suppl. material 3: fig. S5). We observed that isolates from different continents appear to have exchanged partial genomic sequences, indicating that some of the genomes have different phylogenetic origins, which is consistent with gene flow and recombination between diverged lineages (i.e. genetic introgression). These events are illustrated by the diagonal lines of terminal branches in the densitree cladogram.

Isolation-by-distance (IBD) analyses revealed a positive relationship between genetic distance and geographic distance. The Mantel test revealed a significant correlation between the genetic and geographic matrices (R^2^ = 0.3607, *P* < 0.001). Regression analyses, including models with logarithmic, square root and quadratic transformations, demonstrated a positive relationship, with geographic distance explaining up to 35.80% of the genetic diversification observed (quadratic regression F_2,40142_ = 11196.59, *P* < 0.001, R^2^ = 0.3580) (Suppl. material 3: fig. S6). The observed pattern of IBD at local and global scales is characterised by genetic dissimilarity increasing rapidly at shorter distances due to increasingly less efficient gene flow. Interestingly, it appears to be plateauing or even declining at larger distances. This lack of correlation at larger distances may result from genetic drift acting independently in distant populations. Consequently, these populations might be genetically more similar due to random processes or shared historical events, rather than continuous gene flow. In addition, long-distance gene flow, mediated by human activities such as trade or agricultural practices, may obscure IBD signals at larger scales by introducing genetic connectivity between distant populations.

### ﻿Recombinant analysis

**Figure 2.**
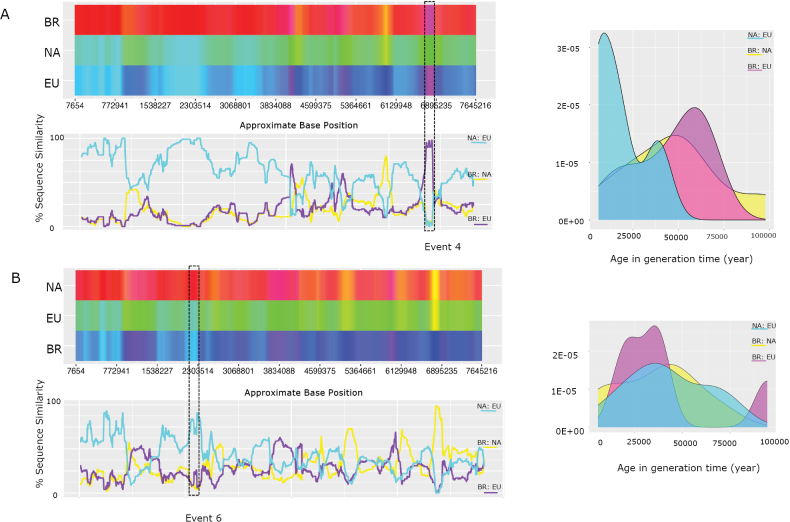
Recombination analysis of chromosome 1. **A** Left: Sequence similarity ﻿visualised through an RBG colour triangle by hybridcheck (involving the isolates BR-85955-2, P-7565-072-8 and I-61851, respectively). The significant recombination event detected by the rpd4 software is enclosed in a dashed box; right: age distribution of recombinant blocks detected by hybridcheck; **B** similar analyses of the BR, EU and NA lineages using the isolates M5.001, CR-34543-1 and A-52621-1, respectively.

Recombination on chromosome 1 was analysed via rdp4, with a focus on fifteen isolates representative of each lineage (North America: LAR318, NRRL13649, I-618151, A-52621-1, CA-CHAT-1; Brazil: BR-85955-2, BR-98290-1, BR-85925-1, M5.001, JAB2; European: F-64330-2, ARG-X5196-1, P-7565-072-8, CR-34543-1, CBS11373). Similar results were found on the other chromosomes (Suppl. material 3: fig. S7). We detected 15 significant recombined blocks with large stretches of nucleotide similarity across lineages (Suppl. material 1: table S5). Amongst the recombination events detected, two (events 2 and 6) were selected for more detailed analysis. We selected these events to illustrate the genomic signature of intercontinental genetic introgression, noting that the other 13 events presented a comparable (albeit less clear) signature. Event 2 involves isolates BR-85955-2 (Brazilian lineage), P-7565-072-8 (European lineage) and I-61851 (North American lineage), henceforth referred to as triplet 1. In addition, event 6 consisted of the isolates M5.001 (Brazilian lineage), CR-34543-1 (European lineage) and A-52621-1 (North American lineage), henceforth referred to as triplet 2. Next, we visualised the recombinant blocks and dated their divergence using hybridcheck (Fig. 2). The visualisation of nucleotide similarities between sequences through an RBG colour triangle revealed a mosaic-like genome structure in these triplets (Ward and van Oosterhout 2016). Rather than a single recombination block as detected by rdp4, the blocks appear to be fragmented, possibly due to subsequent recombination and/or new mutations. The age estimates of those recombinant blocks varied widely amongst blocks (Suppl. material 1: table S6). We observed younger blocks ﻿dating back to 6,100 years before the present and the older block sharing a common ancestor dated to 100,000 years before the present.

We performed an additional recombination analysis to further investigate the variation in introgression age at the intracontinental scale. Isolates from the same lineage were organised into three further triplets: triplet 3 – M5.001:BR-98290-1:BR-85925-1 (Brazilian lineage), triplet 4 – F-64330-2:F-64330-7:F-64330-13B (European lineage) and triplet 5 – NRRL13649:I-61851:CA-CHAT-1 (North America lineage) (Suppl. material 3: fig. S8). The recombinant blocks detected within each lineage were dated using hybridcheck and a Bayesian coalescent approach implemented in beast (Suppl. material 1: tables S6, S7). The results indicated that the number of recombinant blocks and their age estimates differed significantly between lineages (Kruskal–Wallis test, H = 33.5, d.f. = 2, *P* < 0.001). The age estimates provided by hybridcheck revealed that the blocks involving Brazilian and European isolates were younger, i.e. 25,000 and 30,000 years, respectively (Suppl. material 3: fig. S7). In contrast, the North American isolates presented a greater number of blocks, with a much older signature of recombination, with some blocks dating nearly 100,000 years (Suppl. material 1: table S6 and Suppl. material 3: fig. S9).

To apply the Bayesian dating method, we selected three recombination blocks within each triplet identified by hybridcheck (for the blocks selected, refer to Suppl. material 1: table S6, highlighted in yellow). These blocks were aligned against five isolates from each lineage to determine whether they were conserved in the isolates (Suppl. material 1: table S7). Interestingly, these blocks were present in all the isolates, displaying a pairwise identity greater than 99%. These sequences were subsequently used to estimate their age using beast. The dating results were consistent with the hybridcheck algorithm dating results, although there were differences in the order of magnitude of dates between the methods (Suppl. material 1: tables S6, S7). The age estimates for the North American (NA) blocks were more than seven times older than those for blocks from Brazil, ranging from 90,000 to 1,048,860 years (Suppl. material 1: table S7), which was a highly significant difference (Kruskal-Wallis test: H = 32.37; d.f. = 2; *P* < 0.001). This suggests that the NA lineage has experienced more ancient introgression than the other two lineages. We note that, although the age of introgression may be overestimated, for the reasons explained in the Materials and Methods, the relative difference in age estimates is noteworthy and likely to represent genuine differences in the history of introgression between the three geographic clusters.

Additionally, we characterised genes present in the introgressed regions; however, transcripts were recovered only for a few events (Suppl. material 1: table S11). Enrichment analysis for Gene Ontology terms did not identify significant terms within the recombinant blocks. signalp analysis predicted that several proteins correspond to genes encoding secreted proteins (see Suppl. material 1: table S12). However, these regions do not show a significant enrichment for known virulence-associated genes. Therefore, we conclude that genetic exchanges are not likely to occur preferentially in genomic regions associated with virulence.

### ﻿Unravelling population history

**Figure 3.**
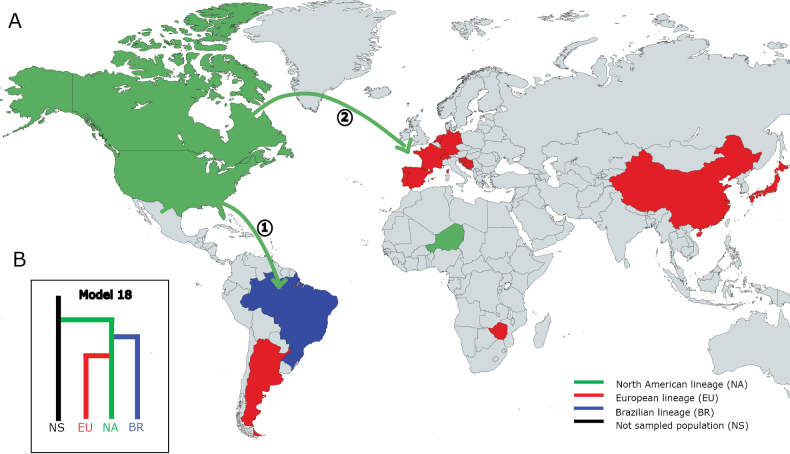
Worldwide sampling of *Colletotrichumgraminicola*. **A** Map showing a likely colonisation route of *C.graminicola* reconstructed by Approximate Bayesian Computation (ABC) using 208 isolates from 17 countries. Different colours indicate distinct lineages and arrows indicate divergence events: **(1)** first divergence event from NA to BR; **(2)** second divergence event from NA to EU; **B** the most strongly supported demographic model was selected based on random forest population voting (see Suppl. material 2 for details).

We analysed the evolutionary history of the isolates, assuming three genetic lineages, North American (NA), Brazilian (BR) and European (EU), as independent populations. In total, we analysed 22 demographic models (Suppl. material 3: fig. S9) using Approximate Bayesian Computation (ABC) based on random forest (RF), conducted in the Python package Pyabcranger (Collin et al. 2020). The demographic models tested were organised into four independent comparisons to determine the most likely ancestral population (Suppl. material 3: figs S10–S12). Overall, our demographic modelling analysis strongly indicates a significant contribution of an unsampled population as the ancestral source shaping the complex evolutionary history of the lineages investigated. However, the North American population appears to have emerged first, followed by the Brazilian and European populations (Fig. 3). North America seems to have acted as an intermediate between the unknown source population and the rest of the world.

### ﻿Virulence analysis

We detected significant differences in virulence amongst the fifty-three isolates evaluated in an analysis of variance test (ANOVA, *P* < 0.0001). Sidäk’s comparison-of-means test revealed that, compared with M1.001, 16 isolates presented significantly greater virulence, whereas 14 isolates presented significantly reduced virulence compared to the reference strain M1.001 (Fig. 1A; Suppl. material 3: fig. S13; Suppl. material 1: table S13). The isolates CBS252.59, MAFF511343 and DMSZ-63127 were non-pathogenic to the maize cultivar evaluated.

The isolates were also classified according to genetic lineage (NA, EU and BR) and sampling year. Differences in virulence between lineages were not statistically significant, according to a Kruskal-Wallis test, although greater variability was observed in the European group (Suppl. material 3: fig. S13a). We used the strain M1.001, which was originally collected from North America in 1972 during the U.S. outbreak and is highly virulent (O’Connell et al. 2012; Rogério et al. 2023), as a control (or baseline) to classify the isolates into three categories (less, equal or more virulent than the isolate M1.001; see Suppl. material 1: table S10). When the isolates were cross-tabulated into these categories, the percentages of strains in the different lineages were statistically significant (Fisher’s exact test, *P* < 0.05). All the isolates from the Brazilian lineage (BR) are less than or equally virulent to the M1.001 strain is, while 41.2% of the European lineage (EU) and 20.0% of the North American lineage (NA) are more virulent. These results indicate a relationship between genetic structure and virulence, particularly in strains that are now more virulent than M1.001, especially in the European lineage. When the isolates were grouped by year (before 2012, between 2012 and 2016 and after 2016), a significant difference was observed between groups (Kruskal–Wallis test, H = 9.02, d.f. = 2, *P* = 0.01). The isolates from 2012–2016 presented the highest virulence, suggesting that, within the three genetic groups, the relative level of virulence can change over time (Suppl. material 3: fig. S13b).

The Mantel test performed between pairwise genetic distance and pairwise similarity in virulence revealed no correlation between these variables (*R* = -0.006257, *P* = 0.47053). Interestingly, the isolates SW-8046-1 and SW-8046-2 exhibited (nearly) identical genotypes. Yet, they displayed contrasting virulence values (Fig. 1A and Suppl. material 3: fig. S14), indicating that minor genetic differences can have a large effect on virulence or that variations in virulence may be attributed to factors other than genetic diversity.

## ﻿Discussion

A global collection of *Colletotrichumgraminicola* field isolates from 17 countries allowed a comprehensive investigation into the evolutionary history of this important maize pathogen. We generated and analysed a broad dataset with a higher resolution than our previous study (Rogério et al. 2023). By including nearly twice the number of isolates, we revealed a consistent global structure, indicating that the three geographically near-isolated populations were restricted and largely defined by their continental origin. We also observed positive correlation in the isolation-by-distance (IBD) analysis at local and global scales. Recombination analysis revealed compelling evidence of genetic introgression between the three geographic groups, suggesting a long history of recombination, possibly through sexual reproduction. The North American lineage displayed the most ancient evidence of recombination. Demographic modelling supported these findings, indicating that North America is an intermediate between the source population and the global distribution, potentially originating from an unsampled population, which is hypothesised to be Mesoamerican. We propose that the global genetic structure of *C.graminicola* is shaped by geographic differentiation driven by long-distance migration and genetic recombination events occurring at distinct points in time.

These lineages exhibit different levels of overall genetic diversity, as indicated by estimated genomic indices, suggesting independent evolutionary trajectories (Suppl. material 1: table S3). The North American lineage possesses the highest estimates of genetic variation. Many factors, including demographic effects can increase genetic diversity. We formulated four (non-exclusive) hypotheses explaining the greater level of diversity in the NA lineage: 1) NA has a larger contemporary effective population size or (2) NA is the ancestral (source) population and other populations experienced a bottleneck when they emerged; (3) The NA is experiencing recurrent incoming gene flow and/or (4) The NA has higher levels of (sexual or parasexual) recombination, limiting the consequences of background selection. Below, we examine these hypotheses in more detail.

Natural gene flow in fungi tends to be mediated through the transport of spores. Members of the genus *Colletotrichum* typically produce asexual spores (conidia) in acervuli immersed in mucilaginous masses disseminated through water splashes, accounting for short-range dispersal (Madden 1997). In contrast, sexual spores (ascospores) are associated with medium- to long-distance dissemination, as they can be ejected upwards and dispersed by air currents, reaching longer distances (Taylor et al. 1999; Alaniz et al. 2019). Sexual reproduction in *C.graminicola* has the potential (even sporadically) to contribute to medium- to long-distance gene flow (Rogério et al. 2023), given the prevailing assumption that asexual reproduction serves as the primary reproductive strategy for short-distance fungal dissemination (Drenth et al. 2019).

Human-mediated transport is a major driver of the long-distance dissemination of plant pathogens, acting as an artificial gene flow mechanism in scenarios where fungal spores may not naturally reach (Finlay 2002). The movement of contaminated seeds and infected plant material, driven by global human trade, can facilitate the migration of pathogens. This artificial gene flow may promote the movement of individuals and their alleles from one population to another, allowing genetic exchange between samples separated continentally, particularly amongst countries with strong trading ties. On a global scale, human-mediated migration is primarily responsible for the geographic differentiation of numerous populations of plant pathogens (Linde et al. 2002; Stukenbrock et al. 2006; Zaffarano et al. 2006; [Bibr B7][Bibr B17]; Goss et al. 2014; Kamvar et al. 2015; McMullan et al. 2018; Tabima et al. 2020; Sotiropoulos et al. 2022).

We detected migration between Argentina and Europe, where isolates from Argentina were grouped within the European lineage. This suggests some form of genetic exchange between these geographically distant regions, facilitated by long-distance gene flow. The role of contaminated seeds as sources of inoculum, promoting the movement of pathogens, has been well established in this pathosystem. In this study, we proposed the hypothesis that winter nurseries in South America, which are frequently employed in breeding programmes, might serve as a potential source for the introduction of the pathogen into new regions. Furthermore, there seems to be a recurring pattern of maize germplasm movement between international breeding companies and experimental stations in Argentina. A case in point is the experimental station “La Josefina S.A”, in Mercedes, Argentina, which provided winter nursey service from 1985–2014, collaborating with numerous (10–15 companies/institutes) maize breeding programmes, including European (Murad 2023, personal communication). This station routinely handles maize seeds from international companies working with germplasm from Europe, the USA and Argentina. Consequently, it regularly receives and returns seeds to the original breeding programmes, potentially contributing to the long-distance dissemination of the pathogen.

We identified signatures of genetic introgression at the genome-wide level between lineages, supported by statistically significant recombination events. The examination of nucleotide similarities between sequences uncovered a mosaic-like genome structure, in which different segments exhibit distinct genetic ancestries. The recombination analysis performed with rdp4 revealed a few large recombinant blocks, which contrasts with the results of hybridcheck which displayed numerous smaller, fragmented blocks. Through visualising nucleotide similarities, we observed a pattern where smaller blocks appeared to be later fragmented by recurrent recombination. Multiple mutations may have accumulated after ancient recombination (i.e. before divergence), giving rise to the observed pattern of fragmented blocks. In essence, these small regions might be remnants of a common ancient introgression event, where subsequent recombination fragmented the originally larger block.

Importantly, subsequent recombination may also increase the genetic divergence between the donor and recipient sequences, making the introgressed regions appear more divergent and hence “older”. We are, therefore, cautious in our interpretation of the actual estimated divergence time dates. Nevertheless, we are confident in our interpretation that the introgression events observed in the North American lineage date further back in time than those in the other two lineages (i.e. Europe and Brazil). Our recombination analysis detected events that took place as recently as 6100 years ago, as well as potentially very old events (> 100,000 years ago). These more recent occurrences are likely indicative of genetic exchanges that have occurred in a relatively recent timeframe if not an ongoing process. Overall, our introgression investigation revealed a prolonged history of genetic exchange.

Sexual reproduction has been observed under laboratory conditions, suggesting an absence of prezygotic reproductive barriers between genetically divergent strains (Rogério et al. 2023) as observed in other plant pathogenic ascomycetes (Gac and Giraud 2008). Although the sexual state of this species has not been documented in nature, our data suggest a significant contribution of genetic recombination resulting from either sexual recombination or other non-meiotic recombination mechanisms, such as parasexual events via hyphal anastomosis, as previously described in other *Colletotrichum* species (Vaillancourt et al. 2000; Souza-Paccola et al. 2003; Roca et al. 2003; Rosada et al. 2010; Nordzieke 2022).

We propose that *C.graminicola* populations may have undergone intense recombination before genetic divergence. Repeated sexual recombination cycles over time could have led to the development of a mosaic-like genome structure, as evidenced by our analyses. Ancient hybridisation events have been observed in the wheat powdery mildew pathogen Blumeriagraminisf.sp.tritici, characterised by admixture between divergent strains resulting in a mosaic genome. Chromosomal segments inherited from both parental sources exhibit fragmented segments, likely produced by backcrossing (Menardo et al. 2016; Sotiropoulos et al. 2022). A similar mosaic-like genome structure has been observed in the oomycete *Albugocandida* (McMullan et al. 2015).

Regarding the evolutionary origin of these lineages, our demographic inference indicates that the North American lineage emerged before the Brazilian and European populations. Age estimates of recombinant blocks detected within lineages further support this finding. Dating the blocks involving isolates from North America revealed that this lineage is older compared to those blocks from other lineages. Moreover, the deeper branches in the phylogenetic tree and the higher level of genetic diversity are all consistent with the North American lineage predating the European and Brazilian lineages.

North America seems to act as an intermediate between the source population and the rest of the world, suggesting a potential colonisation route of the fungus. Our results strongly indicate a significant contribution of an unsampled population as the ancestral source. Given the widely accepted hypothesis that maize domestication originated in Mexico (Matsuoka et al. 2002; Sawers and Sanchez Leon 2011) and that the centre of origin of a pathogenic species usually corresponds to that of its host, the unsampled population might indeed represent a Mesoamerican population. However, our sampling did not include isolates from Mesoamerica, a region that could be linked to the ancestral population. To date, attempts to obtain isolates of *C.graminicola* from Mexico have been unsuccessful and personal communications with researchers have revealed that it is not a disease of major importance in the region.

Recent studies have indicated that maize migrated out of Mexico in two waves at different time points (Wade 2023; Yang et al. 2023). These findings suggest that two (or more) ancestral lineages following the maize dispersal route may have contributed to the genetic diversity of this fungal species. After domestication, maize spread along the Pacific coast, initially reaching Central America (Panama) and then extending through South America, particularly the Andean Region of Peru and potentially Brazil (Kistler et al. 2018). Maize then appears to have moved northwards, reaching south-western North America before reaching Europe (Yang et al. 2023). In Europe, maize was initially introduced into Spain from the Caribbean area in 1494 and then spread through the Mediterranean area, Africa and Asia. Additionally, it was also introduced through the European Atlantic coast from North America (Revilla et al. 1998; Revilla et al. 2022). However, owing to the lack of comprehensive fungal sampling from South America, this occurrence may have originated from the Mesoamerican *C.graminicola* lineage first, given the historical trajectory of the maize movement.

Establishing the origin of emerging infectious diseases is both challenging and important. Identifying the ancestral population reveals the standing genetic variation that could introgress into the outbreak lineages. We encourage future studies to comprehensively survey across the Mesoamerica Region, as well as South America, which would further advance our understanding of how this pathogen has adapted and spread to other parts of the world. A genomic comparison between the genetic variation in the source population and population worldwide would enable the examination of genes and genomic regions that experienced strong selection in invasive strains. Such an analysis could shed light on the role of recombination and hybridisation, which become more powerful when there is a greater presence of ancestral variation.

*C.graminicola* shares several characteristics with another significant maize pathogen, *Setosphaeriaturcica*, which is the cause of northern corn leaf blight. Evidence of sexual recombination in *S.turcica* has been reported in a population genetic study by Borchardt et al. (1998). However, the spread of *S.turcica* in Europe results primarily from the expansion of maize cultivation rather than from rapid adaptation. In this instance, *S.turcica*’s spread seems to be due to clonal reproduction (Vidal-Villarejo et al. 2023), in contrast to *C.graminicola* which is supposed to reproduce sexually. The origin of *S.turcica* appears to be in Mexico, from where it later migrated to North America and then to Europe, following a path similar to that of *C.graminicola* (Borchardt et al. 1998; Miedaner and Garbelotto 2024). Breeding for disease resistance becomes more challenging when dealing with pathogens that undergo recombination. Plant breeders must continuously monitor pathogen populations and develop new resistance genes to keep up with the evolving pathogen strains (McDonald and Linde 2002). In *C.graminicola*, genetic diversity is further enhanced by the existence of three major populations and evidence of migration between them. Increased genetic variation resulting from sexual recombination can drive the rapid evolution of pathogen populations. Breeding strategies may need to incorporate multiple resistance genes or use gene rotation to mitigate the risk of resistance breakdown. Addressing sexual recombination in pathogens requires a proactive and continuous strategy for breeding disease-resistant plants. This underscores the importance of understanding pathogen population genetics and implementing robust breeding strategies to ensure long-term crop protection.

Finally, we observed great variability in virulence among the isolates tested, suggesting potential fungal adaptive evolution. A significant, albeit subtle, relationship was found between genetic groups and virulence levels, with the European group exhibiting increased virulence compared with the strain collected during the U.S. outbreak. This level of virulence changed over time, raising concerns about potential new outbreaks of maize anthracnose, particularly in European cultivation. We found that two pairs of isolates with nearly identical genotypes presented marked differences in virulence, emphasising that adaptive evolution may be attributed to factors other than genetic diversity, such as environmental influences or epigenetic modifications. Additionally, it is well known that minor variations in single genes such as effector encoding genes can have marked effects on virulence. Further studies using these genotypes with contrasting virulence phenotypes may be used to elucidate the genetic basis of virulence in pathogens, contributing to a better understanding of plant diseases and the development of durable control strategies.

## ﻿Conclusion

We have observed two different gene flow patterns, which are likely the result of the natural spread of the pathogen and the exchange of contaminated seeds. Our findings indicate genetic introgression between lineages, revealing a long history of recombination. We have identified significant recombination events occurring at specific points in time, with the North American lineage showing evidence of the most ancient recombination. According to demographic modelling, North America is positioned between Brazil, Europe and an unsampled ancestral source population, which is believed to be Mesoamerican. Our analyses have shown that the global genomic structure of *C.graminicola* is influenced by geographic differentiation driven by long-distance migration and a long history of recombination and introgression.

## ﻿Additional information

### Adherence to national and international regulations

All the fungal strains used in this study have been legally obtained, respecting the Convention on Biological Diversity (Rio Convention).

### Author ORCIDs

Flávia Rogério https://orcid.org/0000-0001-7801-5112

Cock Van Oosterhout https://orcid.org/0000-0002-5653-738X

Stéphane De Mita https://orcid.org/0000-0003-2752-865X

Francisco Borja Cuevas-Fernández https://orcid.org/0000-0002-6543-5094

Pablo García-Rodríguez https://orcid.org/0000-0001-9730-8412

Sioly Becerra https://orcid.org/0000-0003-2564-1982

Silvia Gutiérrez-Sánchez https://orcid.org/0009-0002-4389-4801

Andrés G. Jacquat https://orcid.org/0000-0001-6811-9604

Wagner Bettiol https://orcid.org/0000-0001-7724-6445

Guilherme Kenichi Hosaka https://orcid.org/0000-0001-8353-1138

Rogelio Santiago https://orcid.org/0000-0001-5036-3975

Pedro Revilla https://orcid.org/0000-0002-4826-6073

José S. Dambolena https://orcid.org/0000-0001-8690-6564

José L. Vicente-Villardón https://orcid.org/0000-0003-1416-6813

Ivica Buhiniček https://orcid.org/0000-0002-7743-8954

Serenella A. Sukno https://orcid.org/0000-0003-3248-6490

Michael R. Thon https://orcid.org/0000-0002-7225-7003

### Data availability

The raw Illumina RAD-seq and WGS reads were submitted to GenBank and their accession numbers are listed in Suppl. material 1: table S2.

## Supplementary materials

Supplementary material 1 Supplementary tables Data type xlsx Explanation note **table S1**: Isolates of *Colletotrichumgraminicola* used in this study; **table S2**: Repeated multilocus genotypes removed from the dataset with 212 Colletotrichumgraminicola isolates; **table S3**: Summary of genomic diversity within Colletotrichumgraminicola lineages; **table S4**: Membership proportion of each isolate for *Colletotrichumgraminicola* genetic lineages considering number of cluster (*K*) equal to 3; **table S5**: List of the recombination blocks detected in the RDP4 software under chromossome 1. Table shows the assigned event number, the recombinant and minor parent, the predicted start and end positions each block relative to the referenced contig, and the p values for the different recombination detection methods; **table S6**: Estimated dates of introgression events from triplet1 (BR-85955-2; P-7565-072-8; I-61851), triplet 2 (M5.001; CR-34543-1; A-52621-1), triplet 3 (M5.001: BR-98290-1: BR-85925-1) , triplet 4 (F-64330-2: F-64330-7: F-64330-13), and triplet 5 (NRRL13649: I-61851: CA-CHAT-1) obtained by HYBRIDCHECK algorithm dating; **table S7**: Estimated dates of recombinant blocks detected by HYBRIDCHECK using BEAST software; **table S8**: Description of summary statistics employed on ABC demographic inference; **table S9**: Description of the demographic models tested; **table S10**: Classification of isolate in each genetic group into three categories based on their virulence compared to the control isolate M1.001. Values represent the percentage of isolates in each category; **table S11**: Funtional annotation of genes in the introgression regions detected between Colletotrichumgraminicola lineages; **table S11**: Funtional annotation of genes in the introgression regions detected between Colletotrichumgraminicola lineages; **table S13**: Untransformed virulence data. https://binary.pensoft.net/file/1258064This dataset is made available under the Open Database License (http://opendatacommons.org/licenses/odbl/1.0/). The Open Database License (ODbL) is a license agreement intended to allow users to freely share, modify, and use this Dataset while maintaining this same freedom for others, provided that the original source and author(s) are credited.Flávia Rogério

Supplementary material 2 Supplementary methods Data type docx https://binary.pensoft.net/file/1258065This dataset is made available under the Open Database License (http://opendatacommons.org/licenses/odbl/1.0/). The Open Database License (ODbL) is a license agreement intended to allow users to freely share, modify, and use this Dataset while maintaining this same freedom for others, provided that the original source and author(s) are credited.Flávia Rogério

Supplementary material 3 Supplementary figures Data type docx Explanation note **figure S1**: Manhattan plot of the distribution of the SNPs along the chromosomes; **figure S2**: Population subdivision of *Colletotrichumgraminicola*; **figure S3**: Neighbor-net network showing relationships between isolates of *Colletotrichumgraminicola* identified based on the clone-correct dataset; **figure S4**: Population subdivision on European lineage of *Colletotrichumgraminicola*; **figure S5**: Densitree cloudogram based on 203 samples across chromosomes; **figure S6**: Isolation-by-distance plot showing the correlation between pairwise genetic dissimilarity and geographical distance (in km); **figure S7**: Recombination analysis across chromosomes for isolates M5.001:CR-34543-1:A-52621- and BR-85955-2:P-7565-072-8: I-61851; **figure S8**: Recombination analysis of chromosome 1 for isolates M5.001:BR-98290-1:BR-85925-1 (triplet 3), F-64330-2:F-64330-7:F-64330-13 (triplet 4) and NRRL13649:I-61851:CA-CHAT-1 (triplet 5); **figure S9**: Age distribution of recombinant blocks between lineages; **figure S10**: Demographic models tested; **figure S11**: The fit of the data used in the ABC analysis; **figure S12**: Results of model choice from ABC analysis; **figure S13**: Pathogenic characterization of *Colletotrichumgraminicola* isolates; **figure S14**: Bar plot showing the distribution of the virulence for the three genetic lineages. https://binary.pensoft.net/file/1258066This dataset is made available under the Open Database License (http://opendatacommons.org/licenses/odbl/1.0/). The Open Database License (ODbL) is a license agreement intended to allow users to freely share, modify, and use this Dataset while maintaining this same freedom for others, provided that the original source and author(s) are credited.Flávia Rogério
