# Supplementary material for: ﻿Long-distance gene flow and recombination shape the evolutionary history of a maize pathogen
Source: IMA Fungus. 2025 Feb 21;16:e138888. doi: 10.3897/imafungus.16.138888 (PMC11882024; doi:10.3897/imafungus.16.138888)
Supplement: Supplementary material 3 — Supplementary figures [file imafungus-16-e138888-s003.docx]

**Long-distance gene flow and recombination shape the evolutionary history of a maize pathogen**

Flávia Rogério^1,11^, Cock Van Oosterhout^2^, Stéphane De Mita^3^, Francisco Borja Cuevas-Fernández^1^, Pablo García Rodríguez^1^, Sioly Becerra^1^, Silvia Gutiérrez Sánchez^1^, Andrés, G., Jacquat^4^, Wagner Bettiol^5^, Guilherme Kenichi Hosaka^6^, Sofia, B. Ulla^4^, Jürg Hiltbrunner^7^, Rogelio Santiago^8^, Pedro Revilla^8^, José S. Dambolena^4^, José L. Vicente-Villardón^1,9^, Ivica Buhiniček^10^, Serenella A. Sukno^1*^, Michael R. Thon^1*^

The following Supplementary Figures are available for this article:

**Figure S1.** Manhattan plot of the distribution of the SNPs along the chromosomes.

**Figure S2.** Population subdivision of *Colletotrichum graminicola.*

**Figure S3.** Neighbor-net network showing relationships between isolates of *Colletotrichum graminicola* identified based on the clone-correct dataset.

**Figure S4.** Population subdivision on European lineage of *Colletotrichum graminicola.*

**Figure S5.** Densitree cloudogram based on 203 samples across chromosomes

**Figure S6.** Isolation-by-distance plot showing the correlation between pairwise genetic dissimilarity and geographical distance (in km).

**Figure S7**. Recombination analysis across chromosomes for isolates M5.001:CR-34543-1:A-52621- and BR-85955-2:P-7565-072-8: I-61851.

**Figure S8**. Recombination analysis of chromosome 1 for isolates M5.001:BR-98290-1:BR-85925-1 (triplet 3), F-64330-2:F-64330-7:F-64330-13 (triplet 4) and NRRL13649:I-61851:CA-CHAT-1 (triplet 5).

**Figure S9.** Age distribution of recombinant blocks between lineages.

**Figure S10**. Demographic models tested.

**Figure S11.** The fit of the data used in the ABC analysis.

**Figure S12.** Results of model choice from ABC analysis.

**Figure S13**. Pathogenic characterization of *Colletotrichum graminicola* isolates.

**Figure S14**. Bar plot showing the distribution of the virulence for the three genetic lineages.

**Figure S1.** Manhattan plot of the distribution of the SNPs along the chromosomes. Each point represents the position of a SNP on the x-axis. SNP density was calculated per 10kb across chromosomes using vcftools.

**Figure S2.** Population subdivision of *Colletotrichum graminicola*. (a) Cross-entropy as a function of the number of clusters K modeled in snmf analysis of population subdivision. (b) Principal-component analysis (PCA) with a priori geographical knowledge from sampling (by continent). (c) Bayesian information criteria (BIC) indicating the most probable number of genetic groups.

**Figure S3.** Neighbor-net network showing relationships between isolates of *Colletotrichum graminicola* identified based on the clone-correct dataset. highlights indicate “migrant”, i.e., isolates that cluster within the group of isolates from another geographic location. Asterisks indicate pure isolates (*q >* 0.99).

**Figure S4**. The European clade of *Colletotrichum graminicola.* (A) Neighbor-net network showing relationships between European isolates. (B) Principal component analysis (PCA) with *a priori* geographical knowledge from sampling by country. (C) ﻿Scatterplot from discriminant analysis of principal components (DAPC). We excluded samples from countries with few representative isolates (i.e., samples CBS252.59, DMSZ-63127, FBH-76290, P-7565-072-1, P-7565-072-8, SP-36820-5, and CH-TZ-3).

**Figure S5**. Densitree cloudograms based on 203 samples. Thin lines indicate a possible topology involving one sample. The consensus tree is shown in dark blue. Red dots represent samples from the European lineage (EU), blue dots represent samples from the Brazilian lineage (BR), and green dots represent samples from the North American lineage (NA).****

Chromosome 1.

Chromosome 2.

Chromosome 3.

Chromosome 4

Chromosome 5

Chromosome 6

Chromosome 7

Chromosome 8

Chromosome 9

**Figure S6.** Isolation-by-distance (IBD) plots showing the relationship between pairwise genetic dissimilarity and geographic distance (in kilometers). Regression analyses, including linear, quadratic, logarithmic, and square root transformations, demonstrate a positive correlation between geographic distance and genetic dissimilarity. A). Linear regression: F_1,13467_=1750.41, *P*<10^-6^, R^2^=11.5%. B) Quadratic regression: F_2,12366_=3512.9, *P*<10^-6^, R^2^=34.2%. Transformed data are show. C) Logarithmic transformation (log(x)): F_1,13467_=3644.80, *P*<10^-6^, R^2^=21.3%. D) Square Root  Sqrt(x): F_1,13467_=2991.31, *P*<10^-6^, R^2^=18.1%.

**Figure S7**. Recombination analysis across chromosomes. (A) Sequence similarity among the North American lineage (NA), European lineage (EU), and Brazilian lineage (BR), ﻿visualized through an RBG color triangle by hybridcheck, involving the isolates M5.001, CR-34543-1, and A-52621-1 (triplet 2), respectively. (B) Similar analyses of the BR, EU, and NA lineages using the isolates BR-85955-2, P-7565-072-8, and I-61851 (triplet 1), respectively.

A)

**
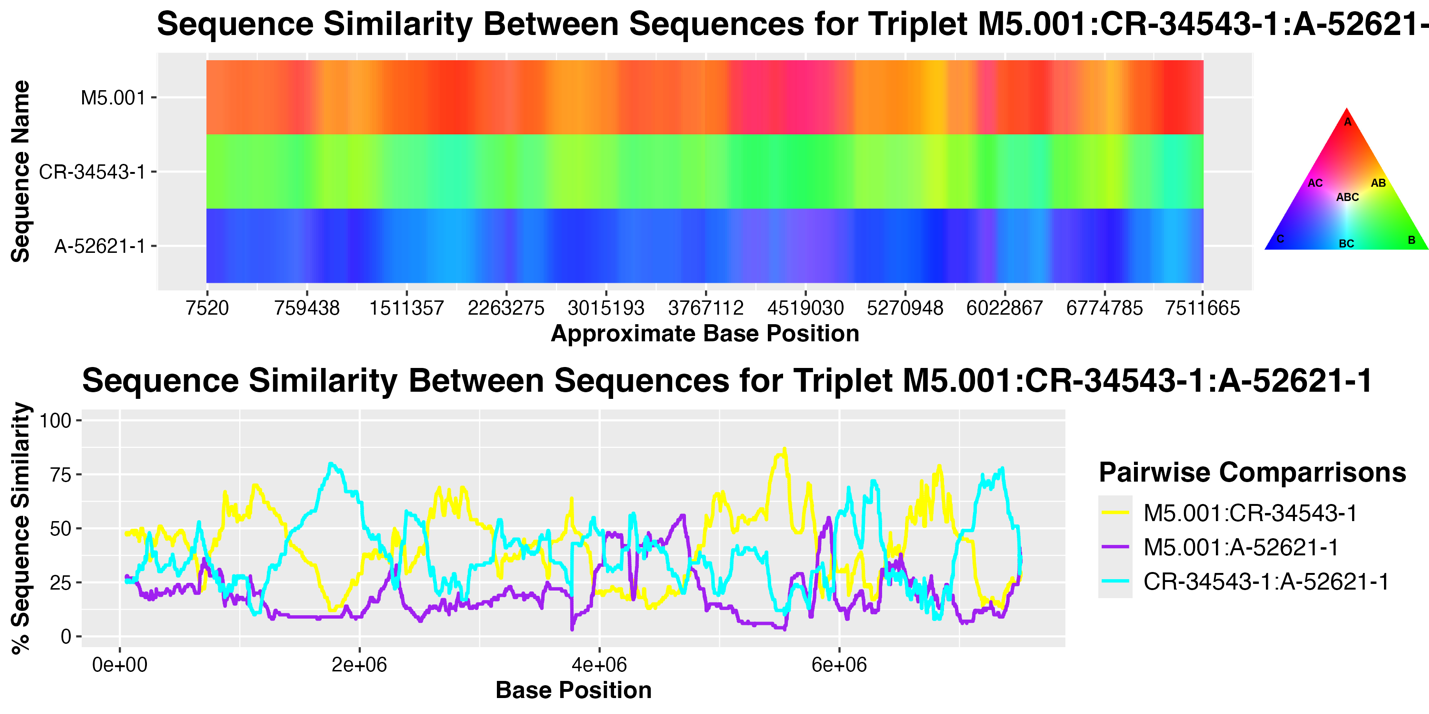
**

B)

**
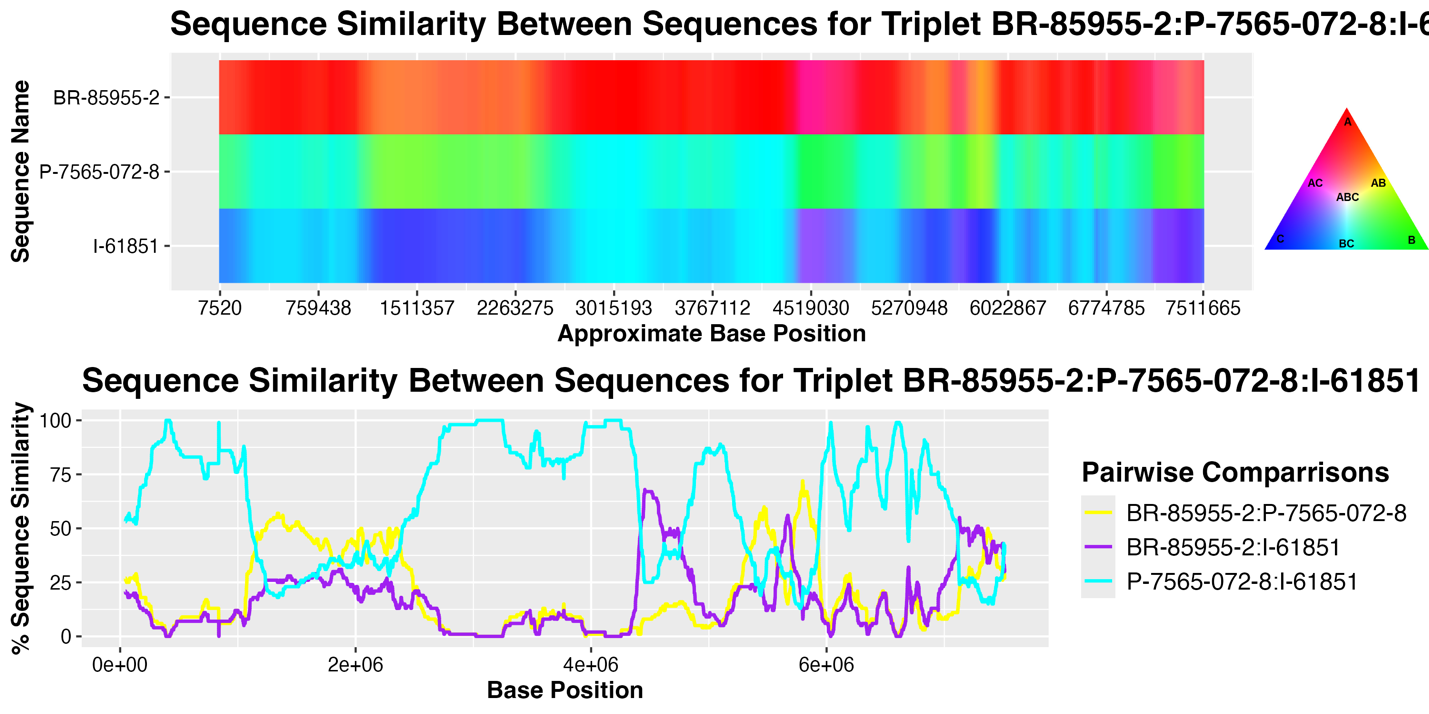
**

Chromosome 2

A)


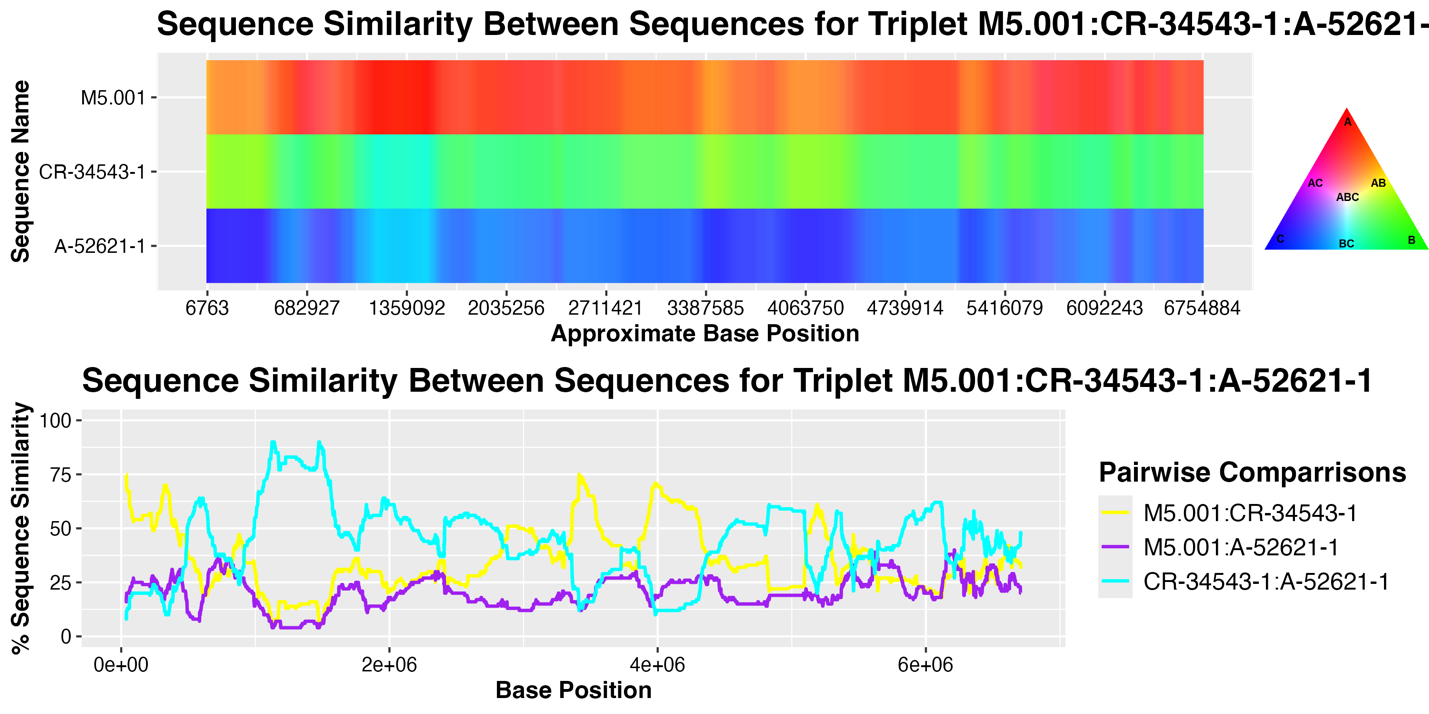

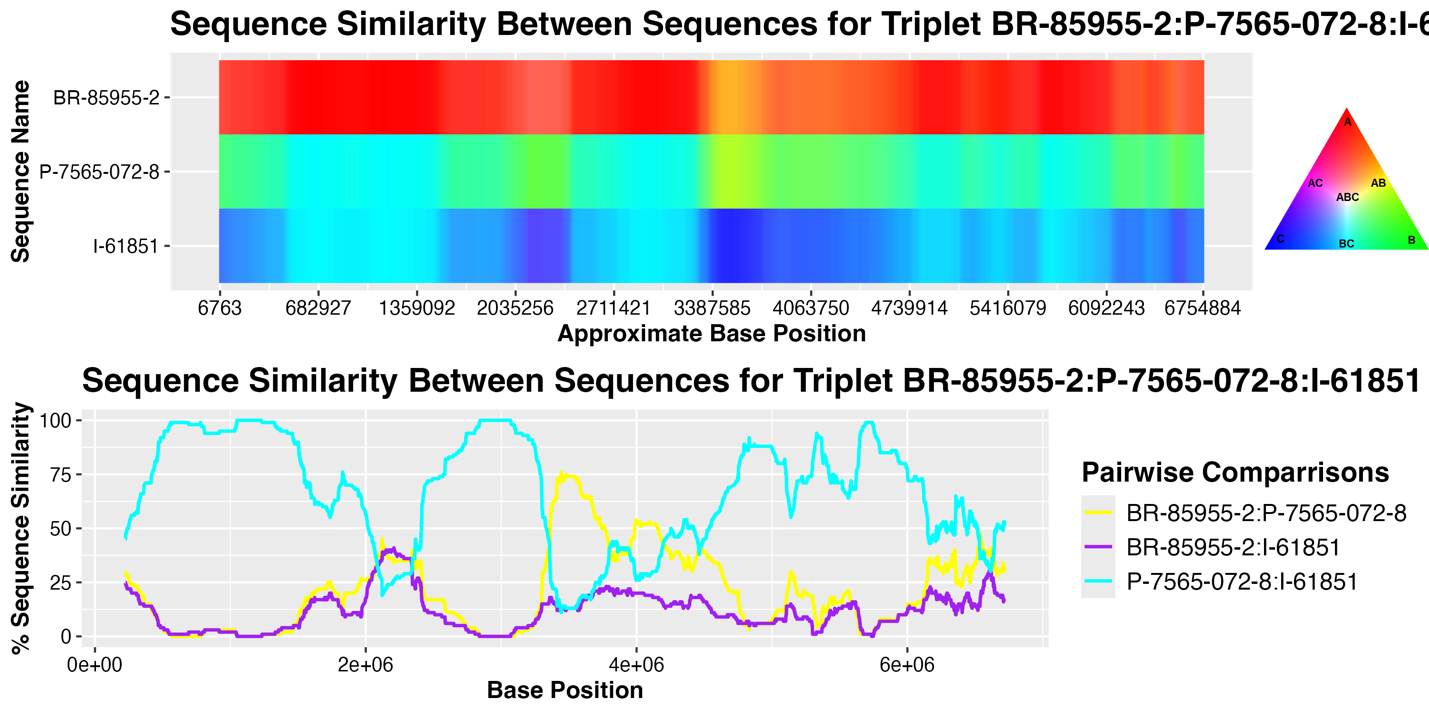


B)

Chromosome 3


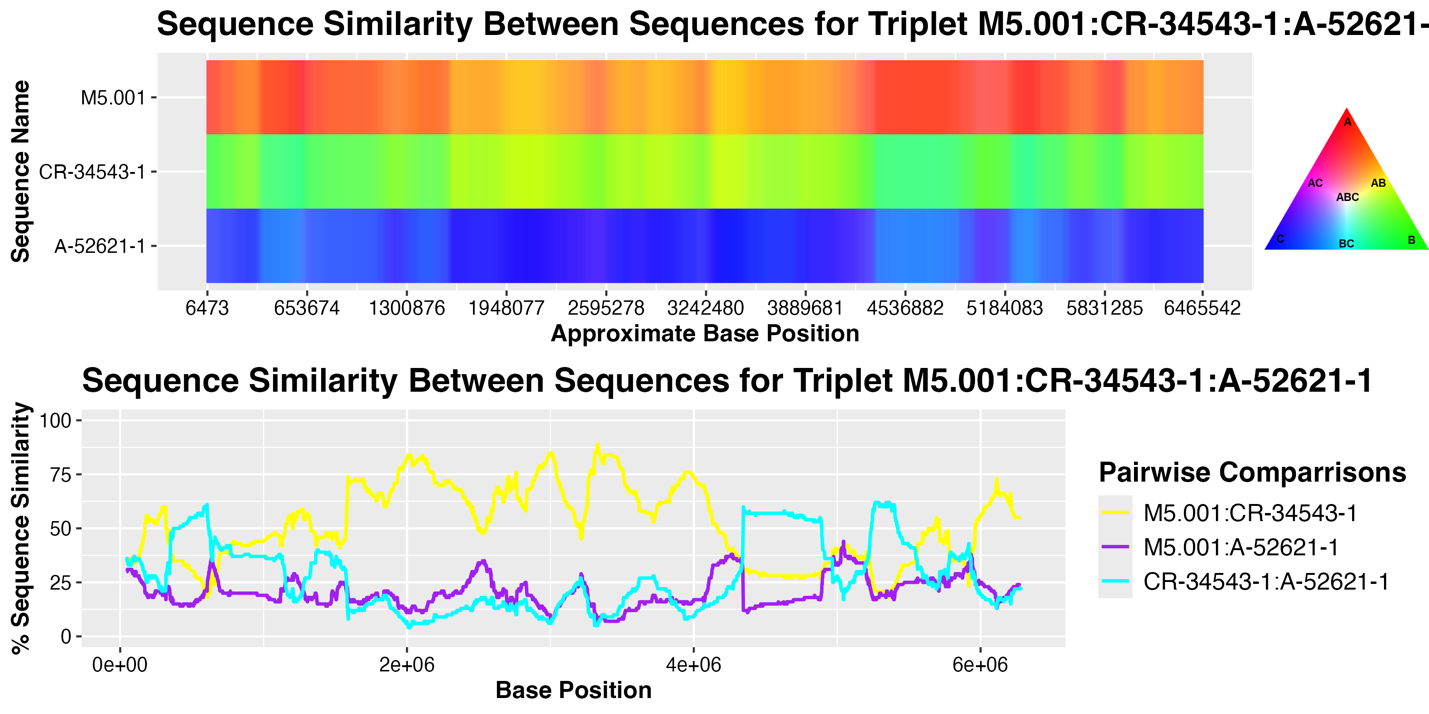

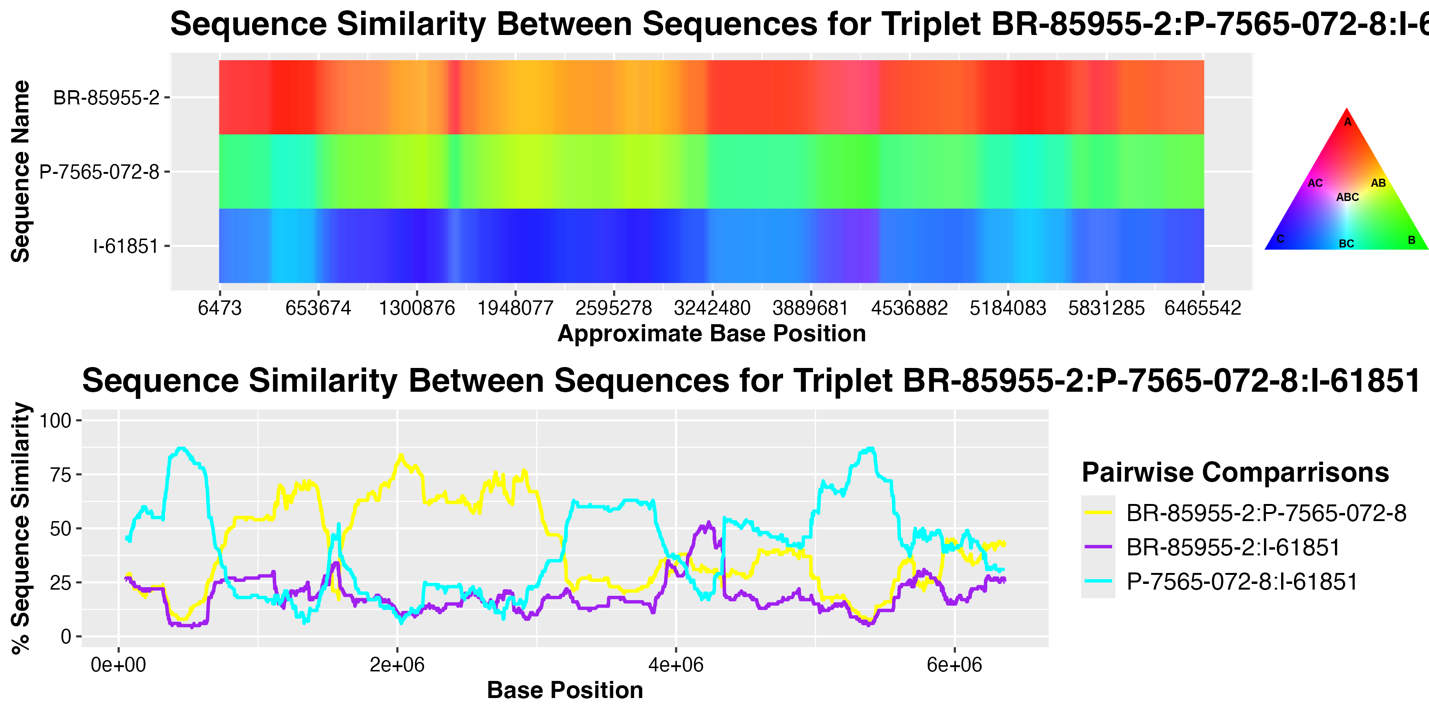


A)

B)

Chromosome 4


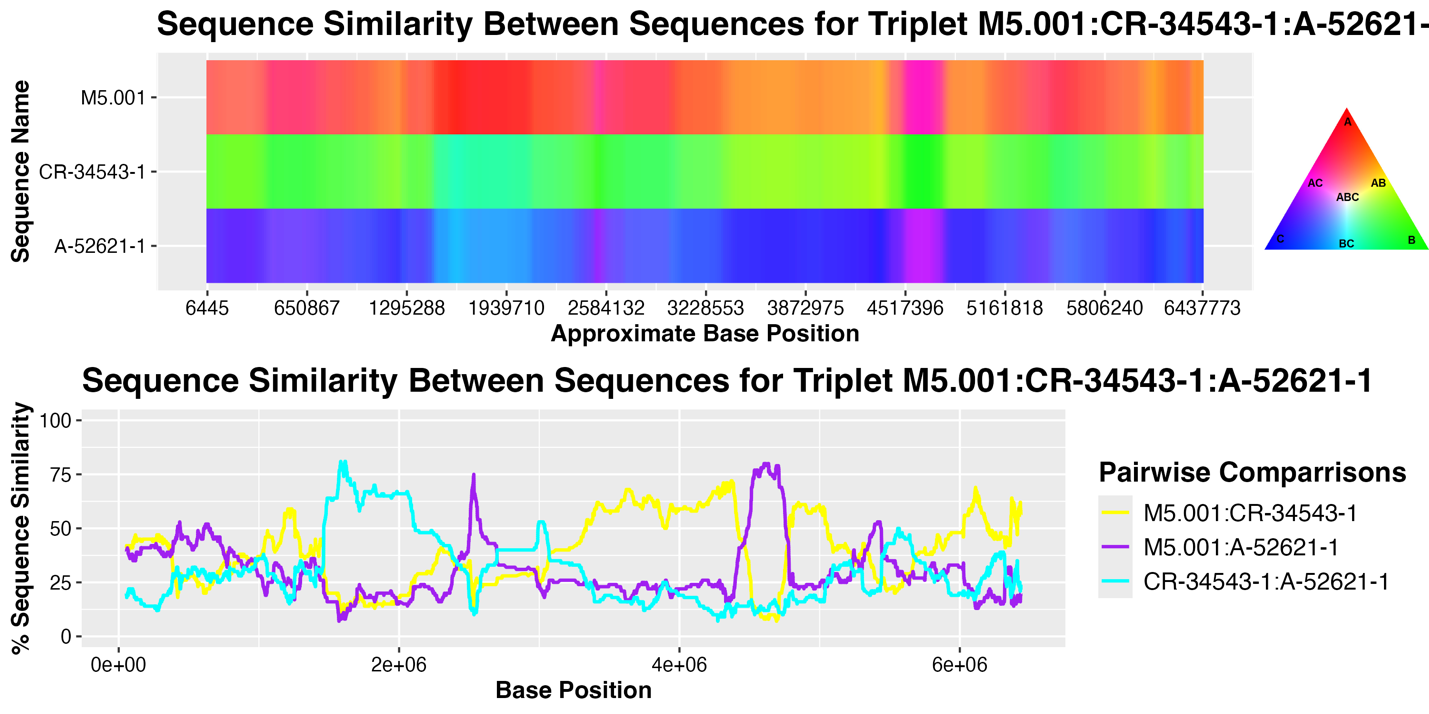

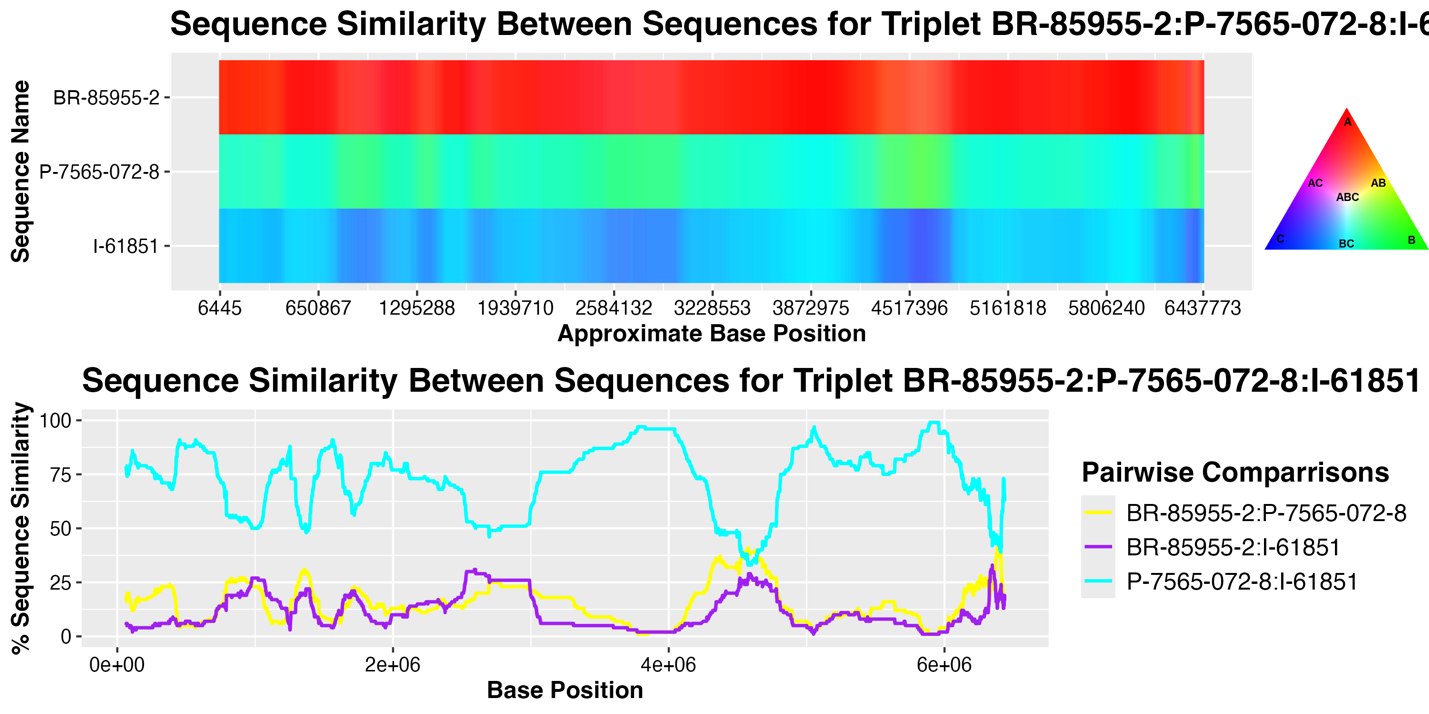


A)

B)

Chromosome 5


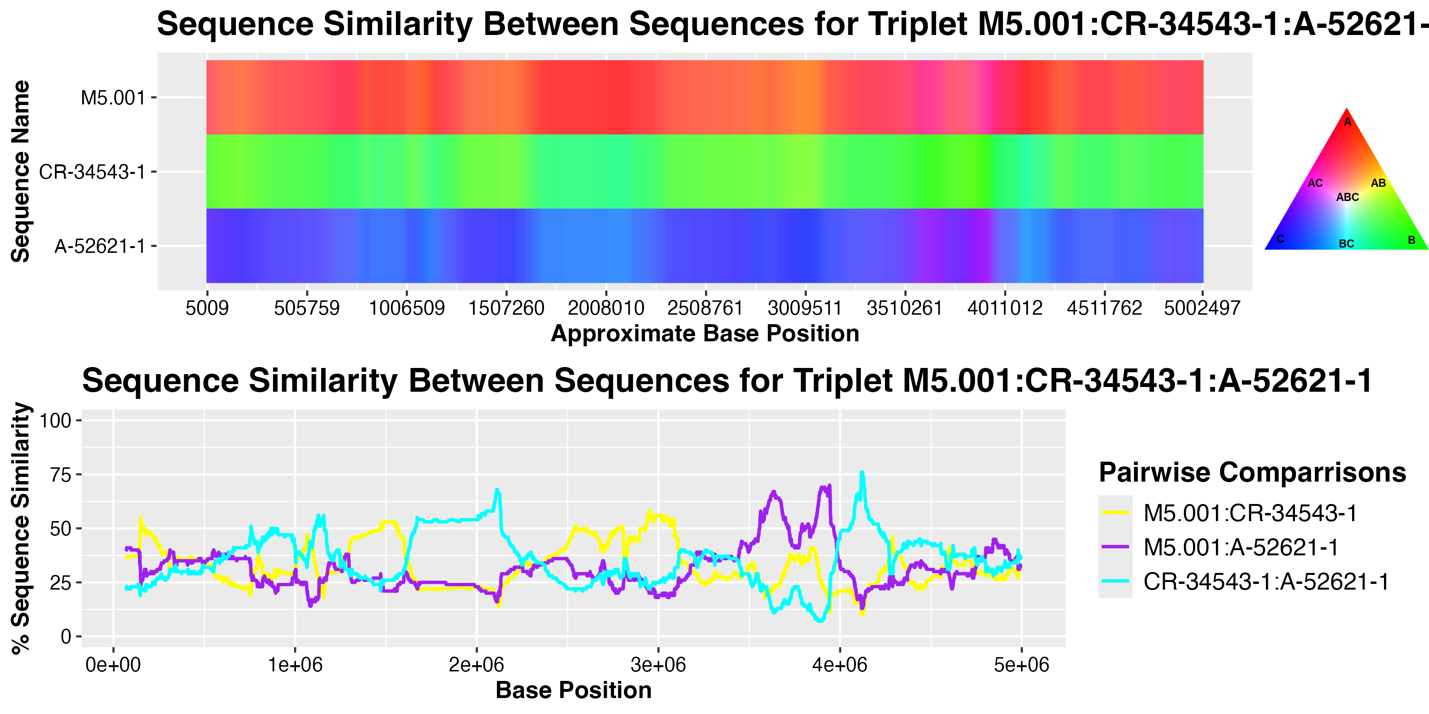

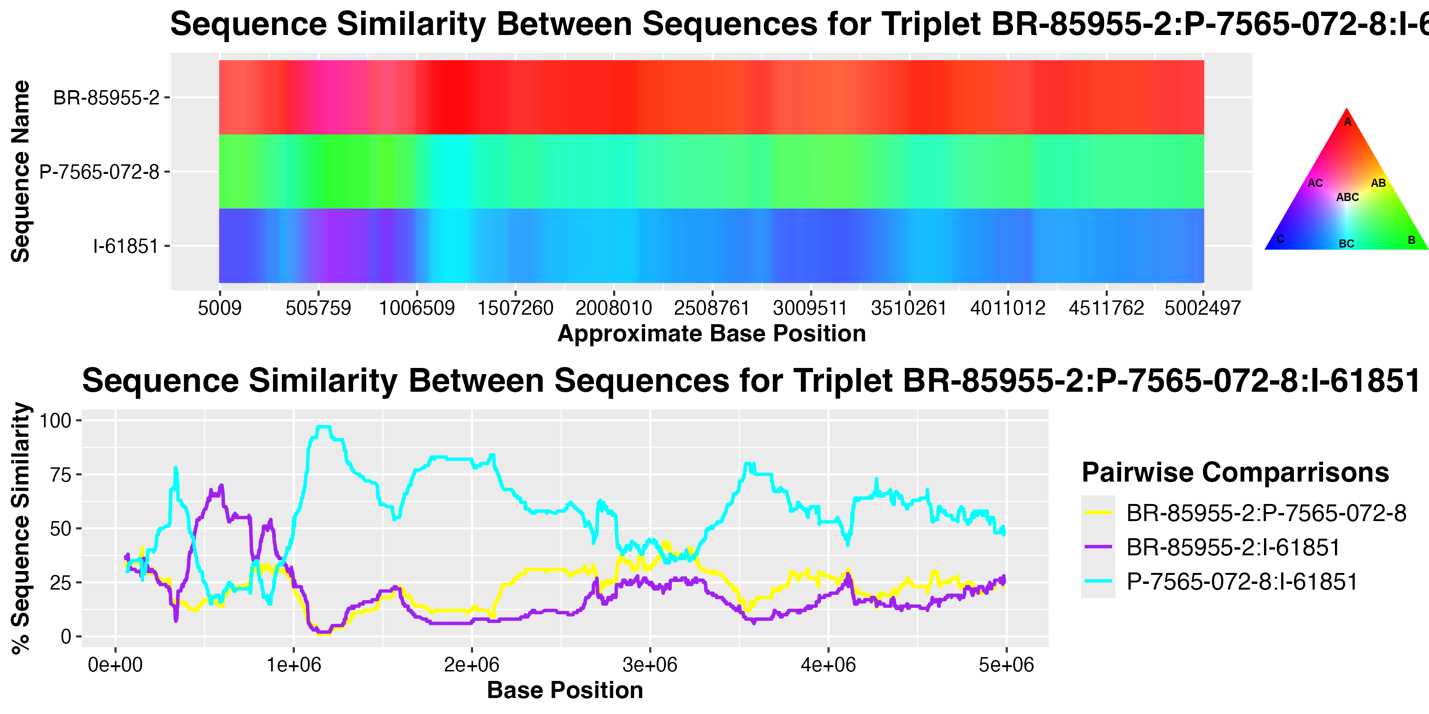


B)

A)

Chromosome 6


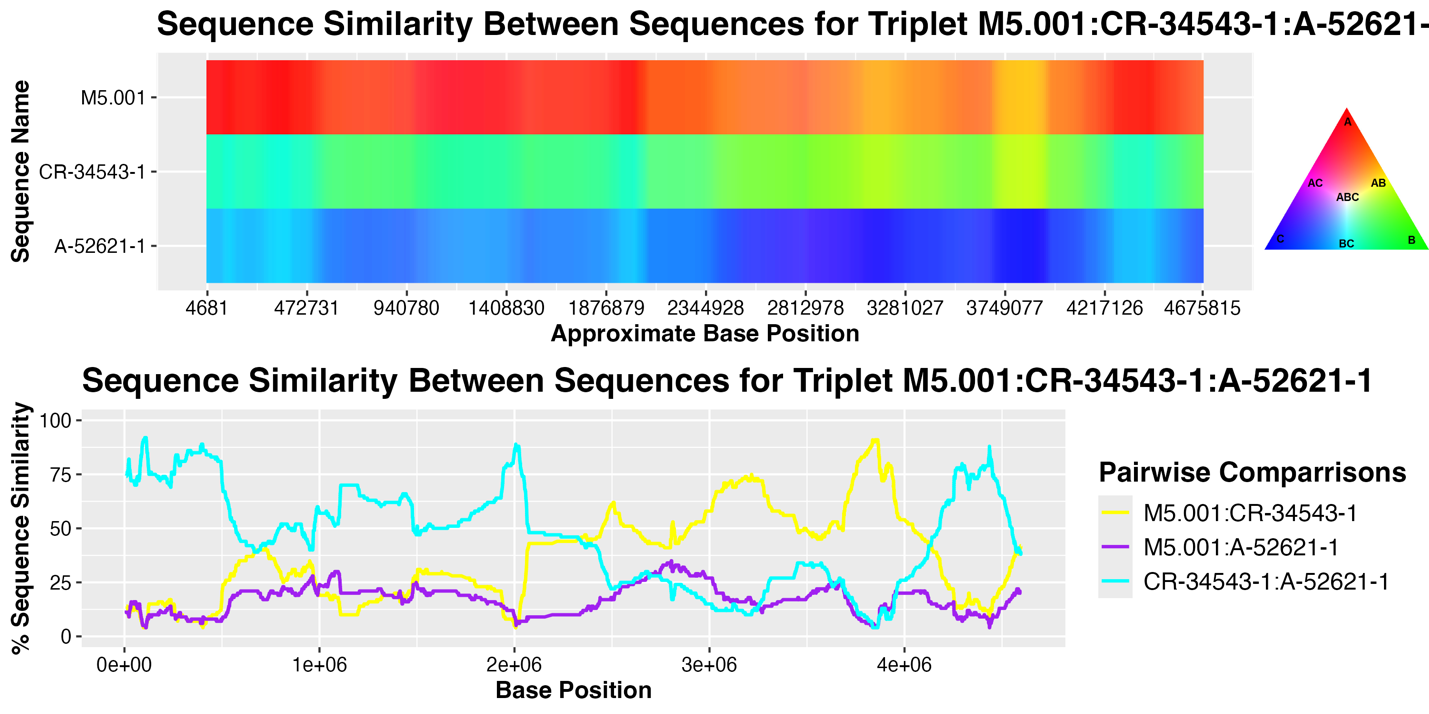

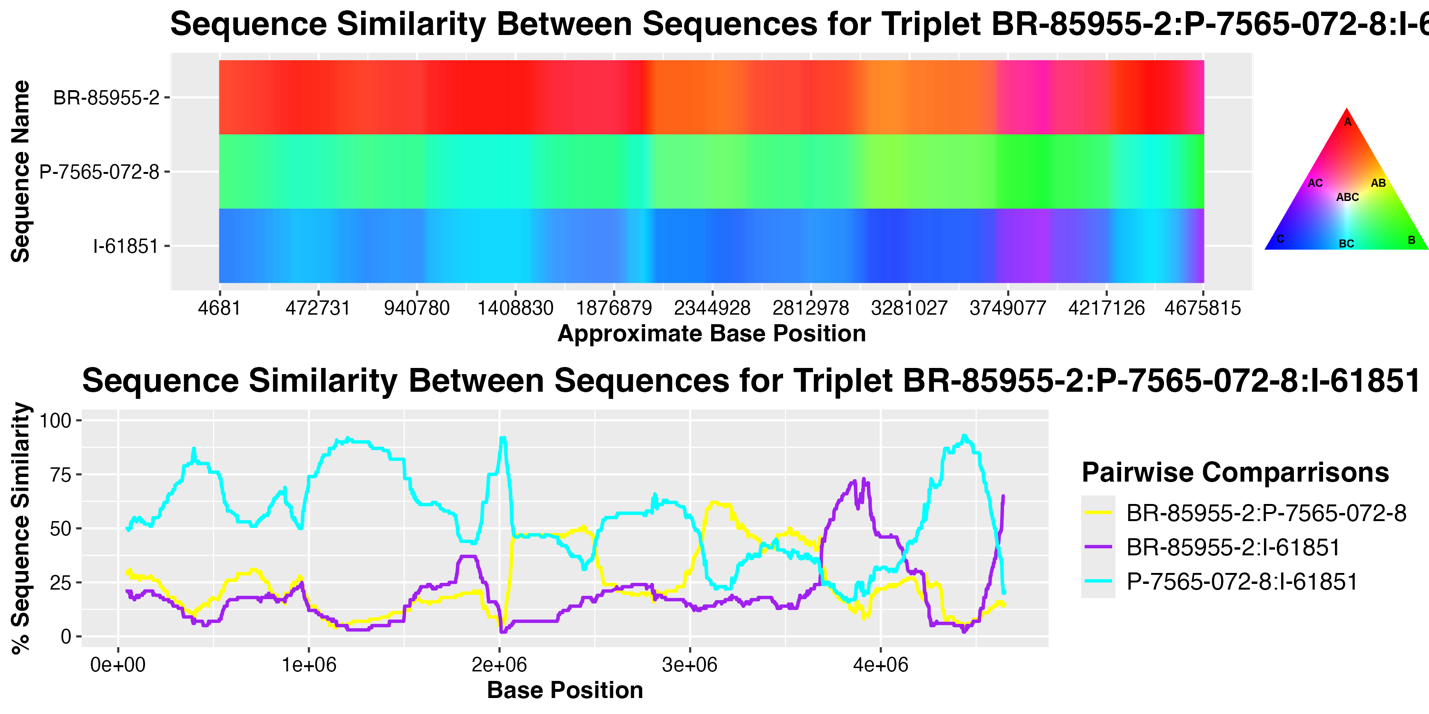
 Chromosome 7

A)

B)


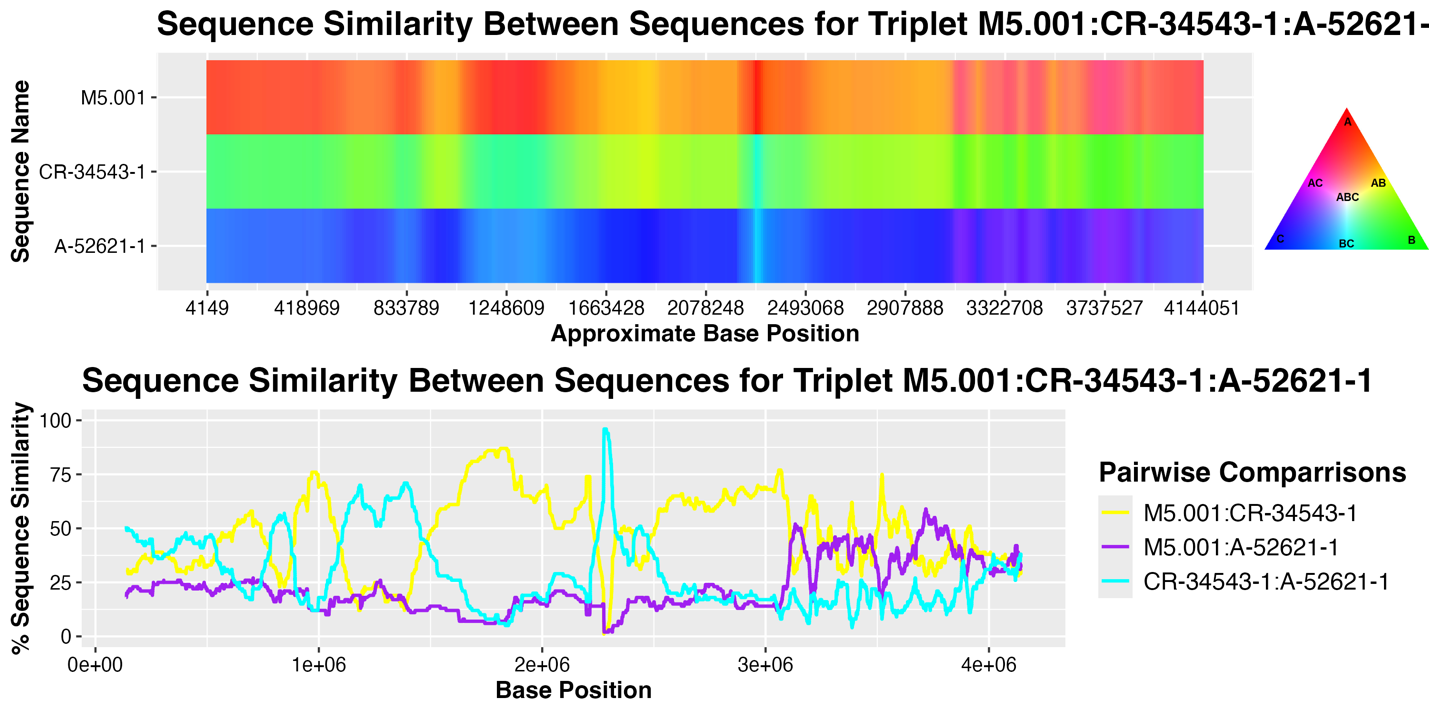

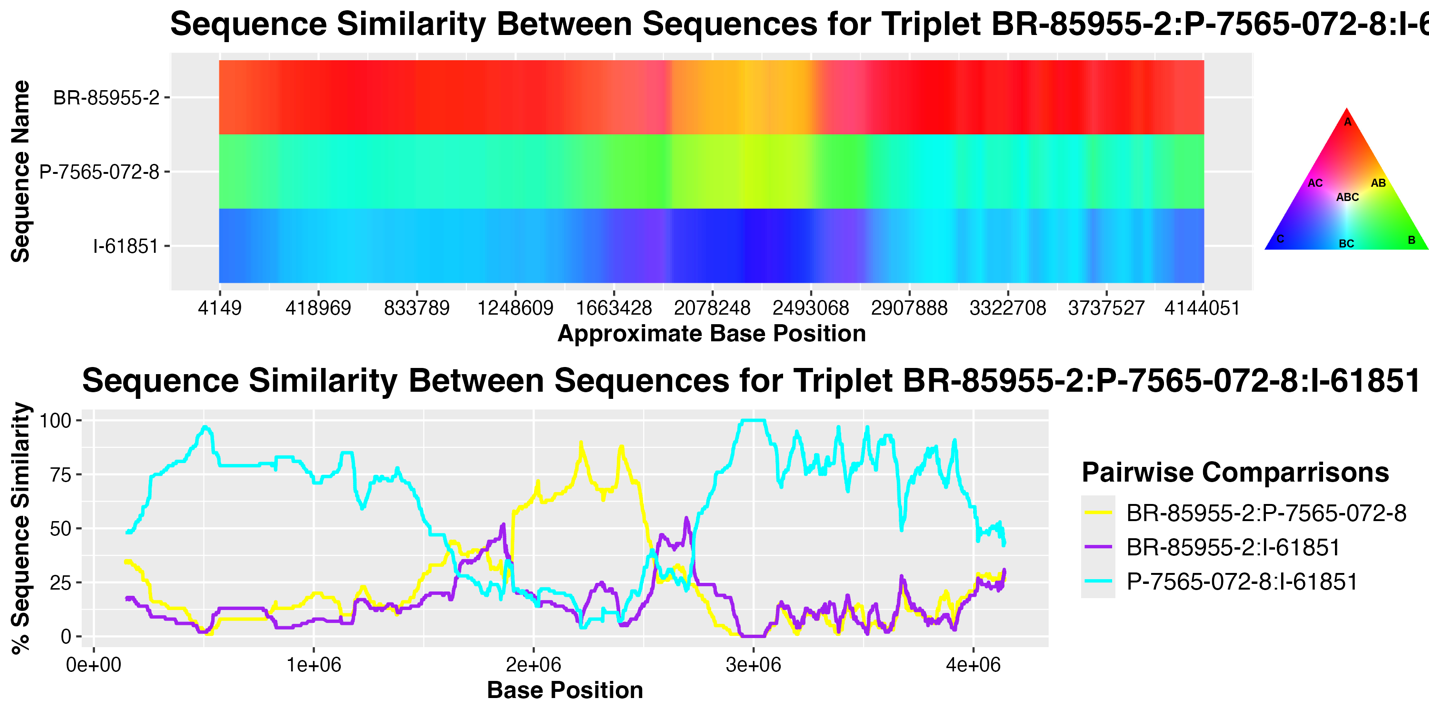
 Chromosome 8

A)

B)

A)


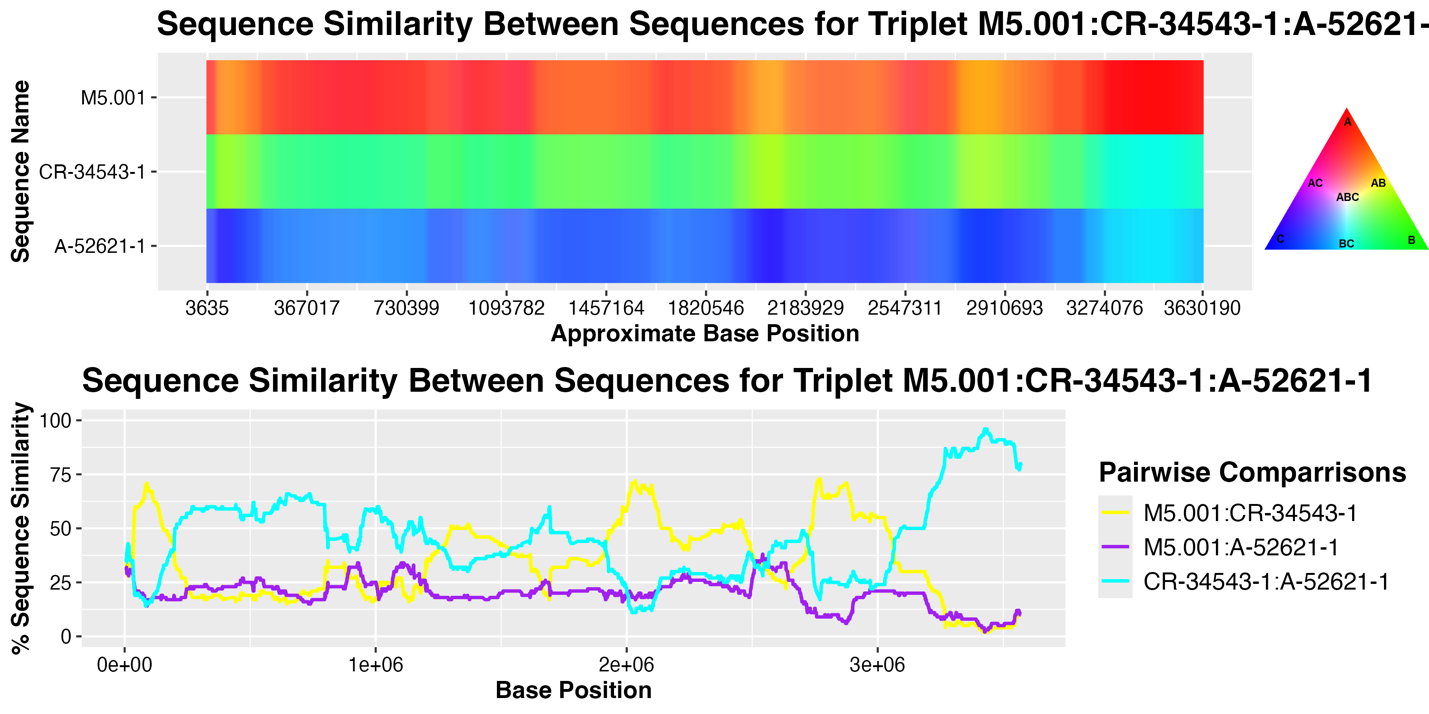

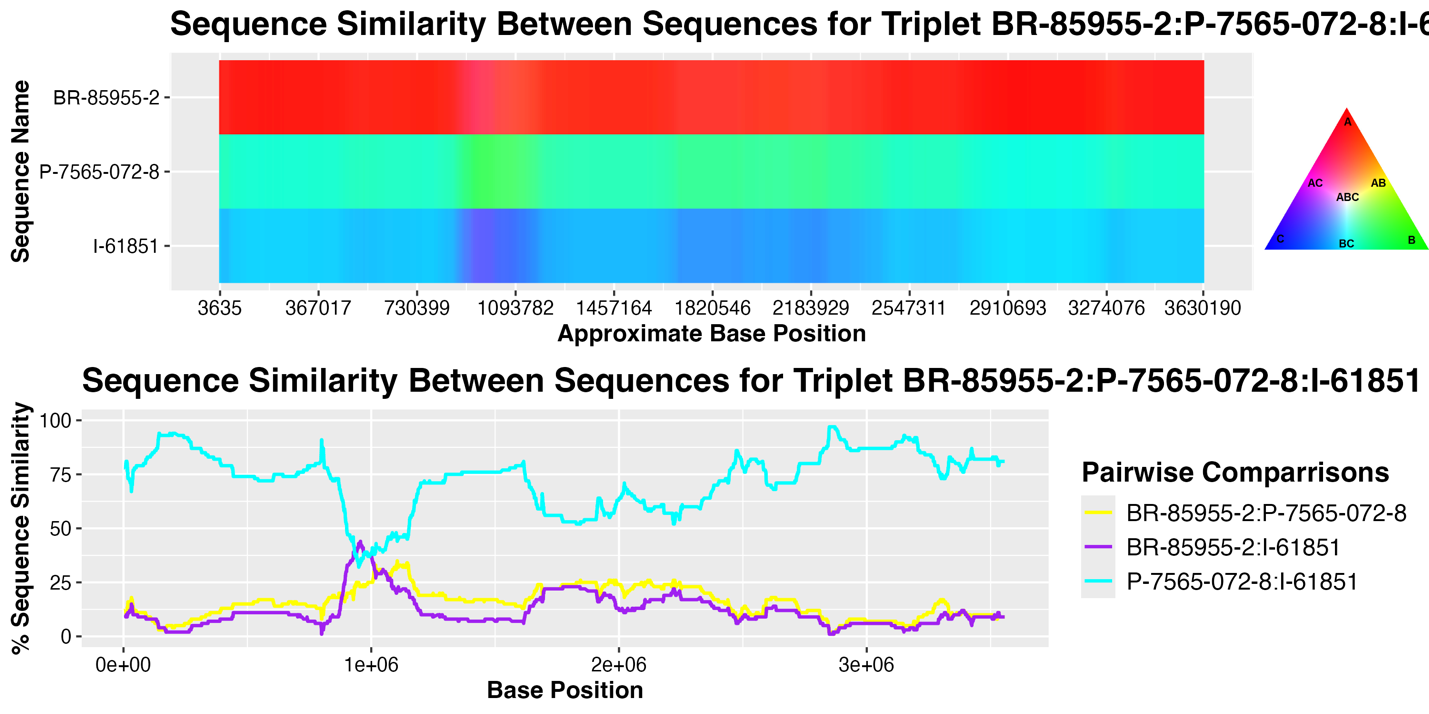
 Chromosome 9

B)


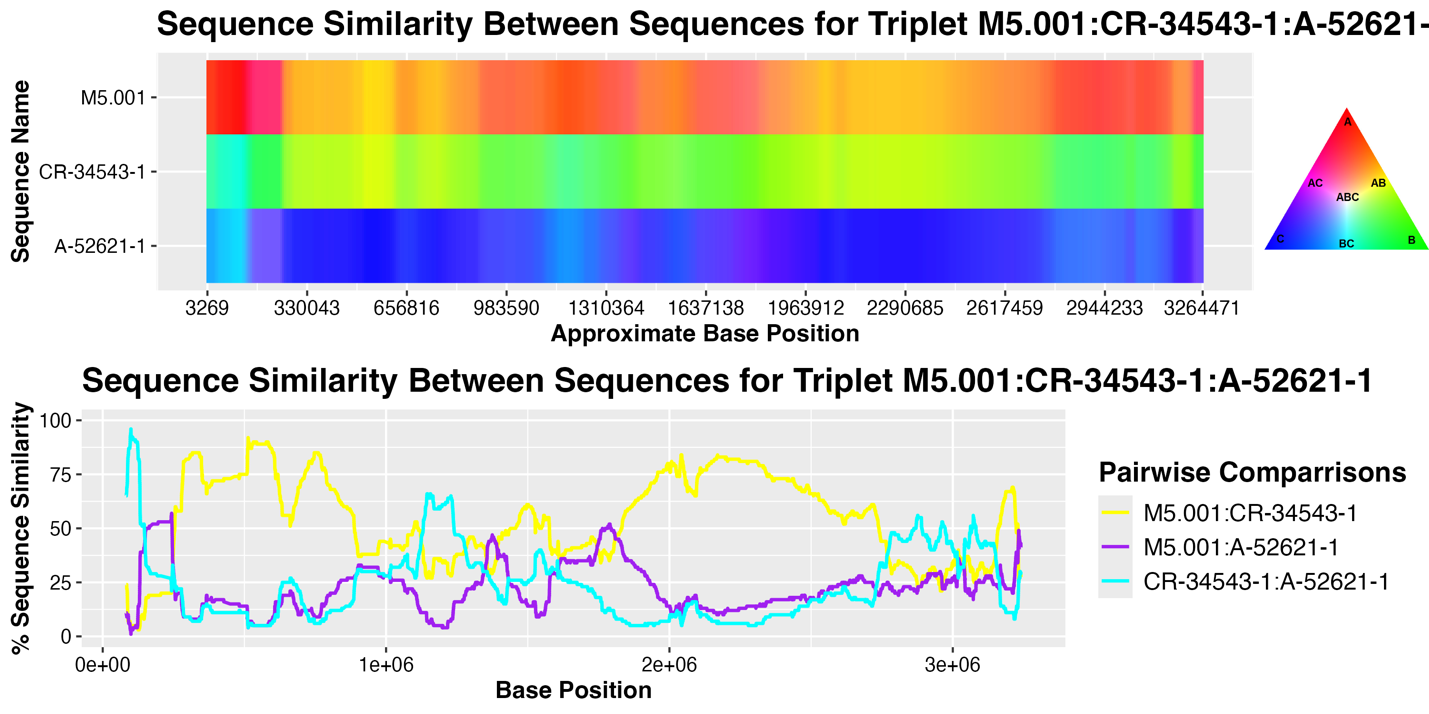

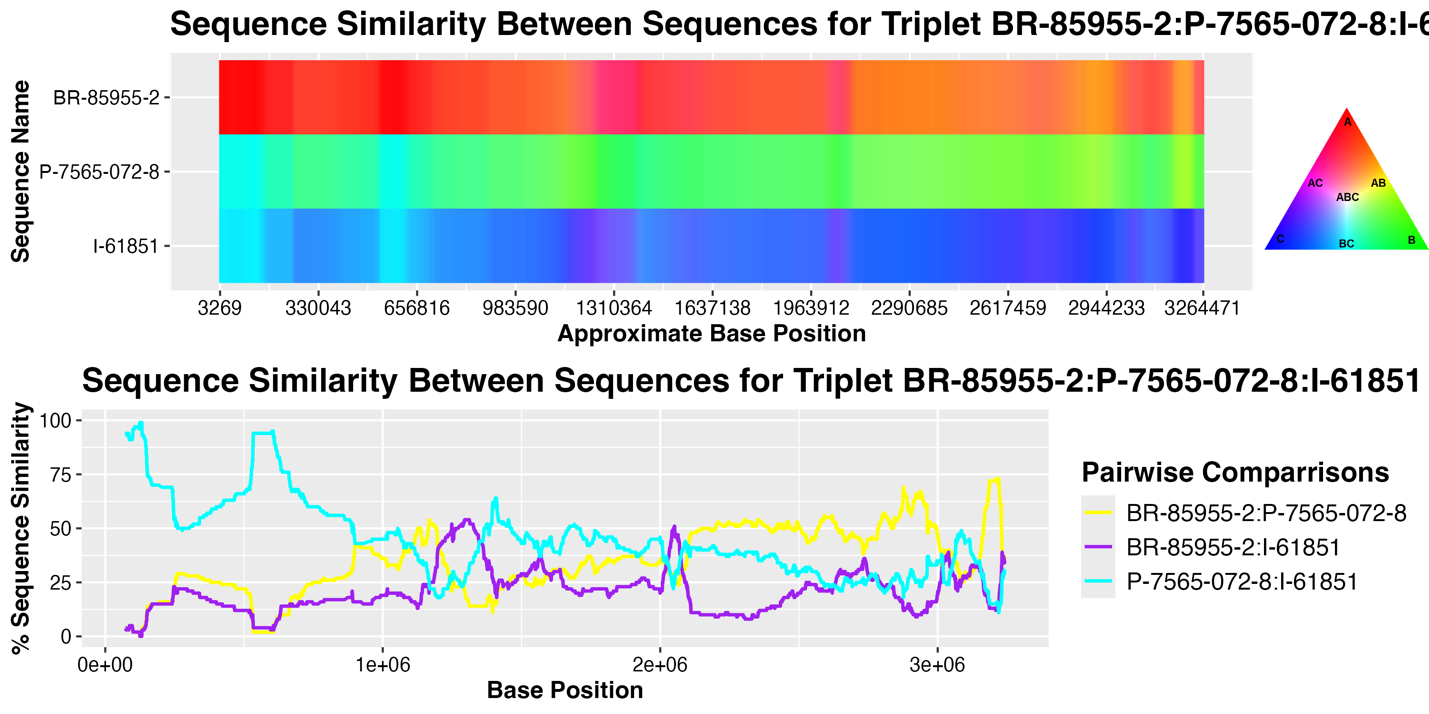
 Chromosome 10

A)

B)

**Figure S8**. Recombination analysis of chromosome 1. (a) Left: Sequence similarity among isolates M5.001:BR-98290-1:BR-85925-1 (triplet 3), visualized through an RBG color triangle by hybridcheck. Right: Age distribution of recombinant blocks detected by hybridcheck. (b) Similar analyses using the isolates F-64330-2:F-64330-7:F-64330-13 (triplet 4). (c) Similar analyses using the isolates NRRL13649:I-61851:CA-CHAT-1 (triplet 5).

**Figure S9.** Age distribution of recombinant blocks involving Brazilian isolates: M5.001:BR-98290-1:BR-85925-1 (triplet 4), European isolates: F-64330-2:F-64330-7:F-64330-13B (triplet 4), and North American isolates: NRRL13649:I-61851:CA-CHAT-1 (triplet 5). Estimates were generated using hybridcheck assuming a mutation rate of 10^−8^ per generation and a generation time of one year.

**Figure S10**. Demographic models tested. Colors indicate the genetic lineage of *C. graminicola* and unsampled ancestral lineage. Rows indicate population divergence.

**Figure S11.** The fit of the data used in the ABC analysis of the population history of *Colletotrichum graminicola* using linear discriminant analysis (LDA). Colored dots indicate simulated data from 30 models tested and the star indicates the observed dataset**.**

**Figure S12.** Results of model choice analysis. Top: number of votes obtained in the random forest for each model or group of models. Bottom: posterior probability of the best model or group of models. The comparisons are replicated over a range of simulations to assess the consistency of the results.

**Figure S13**. Pathogenic characterization of *Colletotrichum graminicola* isolates. (A) Graphic representation of 53 isolates in order of virulence (relative to the reference strain M1.001) in ascending order. Pathogenicity assay batch number is shown in parentheses. A confidence interval using the common estimate of the variability and a Sidäk’s correction is shown around the mean virulence of each strain. Two strains are significantly different if their confidence intervals do not have points in common. Isolates marked with an asterisk were significantly different (*P* < 0.05) from strain M1.001 based on a post hoc test using a Sidäk’s correction. (B) Necrotic lesions on maize leaves at 4 days after inoculation with spore suspensions of the isolates. Black dots indicate the inoculation points.

**Figure S14**. (a) Bar plot showing the distribution of the virulence for the three genetic lineages. (b) Bar plot showing the distribution of the virulence when the isolates were grouped by year.
